# Clinical Management of Orthodontic Miniscrew Complications: A Scoping Review

**DOI:** 10.3390/dj13120582

**Published:** 2025-12-05

**Authors:** Cristina del Rosso, Pier Paolo Poli, Martina Ghizzoni, Alberto Caprioglio

**Affiliations:** 1 Maxillofacial Surgery and Dental Unit, Fondazione IRCCS Cà Granda Ospedale Maggiore Policlinico, 20122 Milan, Italy; pierpaolo.poli@unimi.it (P.P.P.); martina.ghizzoni@unimi.it (M.G.); alberto.caprioglio@unimi.it (A.C.); 2 Department of Biomedical, Surgical and Dental Sciences, University of Milan, Via della Commenda 10, 20122 Milan, Italy

**Keywords:** TADs, orthodontic miniscrew, clinical complications, mechanical complication

## Abstract

**Background**/**Objective**: To outline strategies for the safe clinical use of orthodontic temporary anchorage devices (TADs) by analyzing papers that examine associated risks, complications, and approaches for their prevention and resolution. **Methods**: The research protocol used PubMed, Medline, and Scopus up to May 2024, focusing on controlled and randomized clinical trials aligned with the review objective. Fourteen studies were included; bias risk was assessed, key data extracted, and a descriptive analysis performed. Study quality and evidence strength were also evaluated. **Results**: TADs optimize anchorage control without relying on patient compliance. However, they carry risks and complications. TAD contact with the periodontal ligament or root without pulp involvement requires removal for spontaneous healing. If pulp is involved, the TAD should be removed and endodontic therapy performed. If anatomical structures are violated, TAD should be removed. If transient, spontaneous recovery occurs, but sometimes pharmacological treatment may be needed. A 2 mm gap between the TAD and surrounding structures can prevent damage. In the maxillary sinus, a less than 2 mm perforation of the Schneiderian membrane recovers spontaneously; wider perforations require TAD removal. Good oral hygiene and TAD abutments prevent soft tissue inflammation, which resolves with 0.2% chlorhexidine for 14 days. Unwanted forces can cause TAD fractures, requiring removal. Minor TAD mobility due to loss of primary stability can be maintained; significant instability requires repositioning. **Conclusions**: The use of TADs requires meticulous planning, radiological guidance, and monitoring to minimize risks and manage complications. With proper care, TADs improve orthodontic outcomes and patient satisfaction.

## 1. Introduction

Temporary anchorage devices (TADs), particularly miniscrews, have revolutionized orthodontics by providing reliable skeletal anchorage without relying on patient compliance. TADs are fixed to bone to support or eliminate reactive units and are removed after use [1]. They address the critical challenge of anchorage control, enabling controlled tooth movement and reducing the limitations of traditional methods [2,3,4,5].

The development of miniscrews was inspired by the need for maximum anchorage with minimal patient cooperation [2,3,6]. In 1983, Creekmore and Eklund demonstrated that small screws could withstand orthodontic forces [7,8,9]. Later innovations, such as Kanomi’s orthodontic mini-implant (1997) and Costa’s bracket-like miniscrew (1998), made TADs smaller, simpler to insert and remove, and capable of immediate loading [7].

Compared to traditional implants, miniscrews are minimally invasive, do not require osteointegration, have immediate load-bearing capability, and can be placed in constrained anatomical areas due to their smaller size and smoother surfaces [2,3,5,10]. They are versatile and can be used in various procedures such as space closure, molar uprighting, open bite correction, correction of canted occlusal planes and dental midline alignment, molar distalization or mesialization, anterior retraction, and teeth extrusion or intrusion [2,7,10,11,12]. Miniscrews are especially useful in cases in which conventional anchorage techniques are impractical such as in edentulous spaces, the palate, the zygomatic process, or the retromolar region [13].

Despite their benefits, miniscrews have some limitations. Contraindications include compromised systemic health, poor oral hygiene, pathological bone quality, and smoking [2,12]. TADs failure can arise from factors such as root or periodontal ligament contact, inadequate insertion torque, soft tissue inflammation, and anatomical complications such as sinus perforation or nerve injury [2,12,13,14]. The mandible shows higher failure rates due to increased bone density and insertion torque requirement [2].

Proper screw design and insertion technique have a key role to ensure treatment success. Screws may be self-tapping or self-drilling, with cylindrical or conical shafts and diameters ranging from 1 to 2 mm [2,7,14]. Thinner screws reduce the risk of root contact but are more prone to fracture, especially in areas of high bone density [2,7]. The choice of insertion site influences screw design; for example, thicker cortical bone may necessitate shorter screws, while thinner bone requires longer devices to achieve stability [2,7]. Other anatomical factors related to the insertion technique should also be contemplated. In the maxilla, miniscrews should be inserted obliquely at a 30–40° angle, while, in the mandible, a parallel or slightly oblique (10–20°) insertion is recommended [12,13]. Pilot drilling may be required for cortical bone exceeding 2 mm in thickness, ensuring miniscrew stability and reducing insertion resistance [7]. The self-tapping method is the most common insertion technique, requiring pilot drilling to ensure precision and mechanical stability. Pilot holes should be 0.2–0.5 mm thinner than the implant diameter [15,16]. The newer self-drilling method simplifies placement by eliminating the need for pilot drilling [17,18]. In cases without digital support, TADs are placed perpendicular to the bone surface behind the third palatal rugae, using clinical landmarks for guidance. Polyether impressions ensure accuracy and dimensional stability. Casts are made with plaster, replicating TAD positions for orthodontic device fabrication [19,20,21,22].

The digital workflow involves CBCT imaging and a superimposed dental scanning of the patient palate and upper arch. Miniscrews are selected from a virtual library and placed based on bone availability and the intended device. Placement typically occurs in the anterior paramedian area, between the second and third palatal rugae, 4–5 mm from the midline, ensuring parallelism and avoiding tooth roots [20,21,22,23]. The surgical guide is digitally designed with guiding holes, prototyped, and finalized. During the procedure, local anesthesia is applied, and a drill with a calibrated stop is used to create pilot holes in the palatal cortical bone. After miniscrew placement, scan bodies are captured in a digital impression, and the orthodontic device is fitted as the final step [24,25]. Miniscrews have proven to be a versatile and reliable tool for anchorage in orthodontics. However, their success depends on careful planning, proper insertion techniques, and effective management of complications. Maintaining good oral hygiene is crucial to minimize soft tissue inflammation and the risk of screw loosening [12,26].

This review aims to investigate the complications and risks associated with orthodontic miniscrews and outline strategies for their prevention and management.

## 2. Materials and Methods

### 2.1. Focused Questions

What are the clinical and surgical complications related to orthodontic miniscrews?

### 2.2. Eligibility Criteria

The inclusion criteria for this review were (I) study design—interventional studies and observational studies; (II) patients undergoing miniscrew placement; (III) interventions—miniscrew placement; and (IV) outcome—clinical and mechanical complications of TADs. The analysis focused solely on studies that met all the inclusion criteria. Studies were excluded if they met any of the following conditions: (I) abstracts published in languages other than English; (II) duplicate publications; (III) studies unrelated to the specific research questions, such as those examining different supplementary treatments or not matching the abstract content; (IV) ex vivo or animal experimental studies; (V) studies lacking ethics committee approval; (VI) narrative, systematic, or meta-analytical review articles; and (VII) case reports lacking sufficient scientific validity compatible with the requirements of our work.

### 2.3. Search Strategy

A three-step search procedure was conducted based on the Joanna Briggs Institute (JBI) methodology for scoping reviews. First, an initial limited search was performed using PubMed (MEDLINE) and Scopus. Next, key terms were gathered from the retrieved articles to develop a comprehensive search strategy. Lastly, the reference lists of all selected articles were reviewed to find any other relevant studies [27].

Additionally, the population–concept–context (PCC) framework was utilized. PCC is based on three key elements: the population (people who experience complications from TADs insertion), the concept (complications related to TADs placement), and the context (not limited to any particular cultural or environmental setting). This review involved careful examination of study abstracts focused on various complications related to TADs placement. Throughout the process, adherence was maintained to the Preferred Reporting Items for Scoping Reviews (PRISMA-ScR) guidelines, as outlined in Table S1 [28].

### 2.4. Research

The terms used for Medical Subject Headings (MeSH) included “orthodontic anchorage procedures”, “dental im-plant”, “intraoperative complications”, “root resorption” “cicatrix”, and “mouth mucosa”. Research was conducted in the PubMed (MEDLINE), Scopus, and Web of Science databases.

Articles published from 2004 to 2025 were included, and data was collected between February 2024 and May 2025.

The search was carried out by two reviewers (M.G. and C.D.R.). Any disagreements that arose during the review process were settled through mutual agreement among the reviewers. An additional reviewer (P.P.P.) was consulted for complex cases. The first step of screening consisted of reviewing article titles and abstracts to eliminate those that were not relevant. Afterward, the selected articles were examined in detail by thoroughly reading their full texts. The results were documented, and studies that met the established inclusion criteria and showed similarities were included in this review. This protocol has been registered on the Open Science Framework platform (Registration DOI: https://doi.org/10.17605/OSF.IO/QSKXF, accessed on 21 June 2025).

The specific search strategies used for each electronic database are detailed in Table S2.

### 2.5. Quality Assessment of Included Studies

The possibility of bias in clinical studies was assessed in this research by using a qualitative method based on the National Heart, Lung, and Blood Institute (NHLBI) Quality Assessment tool for Controlled Intervention Studies applicable to Observational Cohort and Cross-Sectional Studies. This approach provided a detailed and organized evaluation of the quality and bias risks in the studies included, aiming to confirm the reliability and validity of their findings [29].

## 3. Results

**Figure 1:**
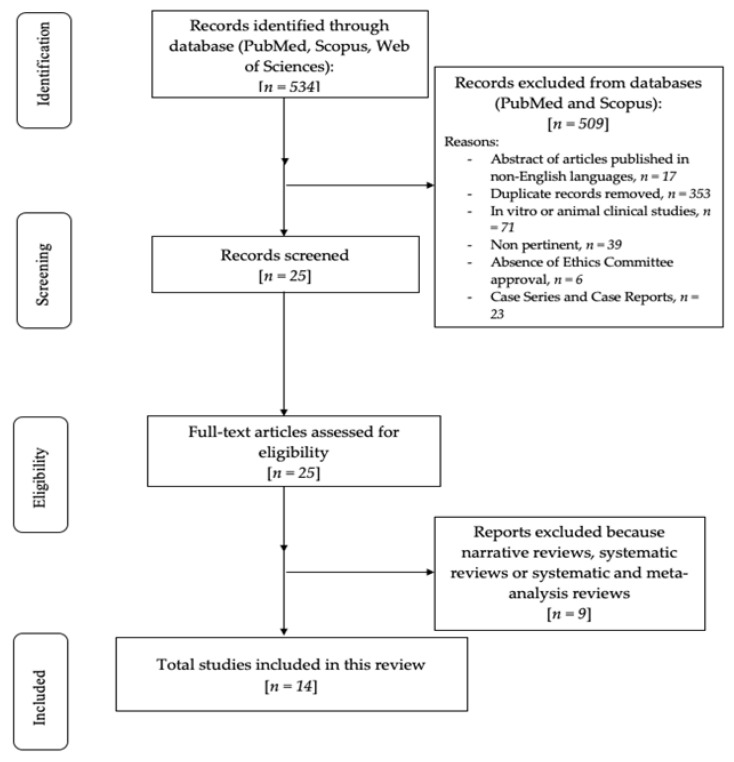
Flowchart of the review process.

The preliminary search selected 534 articles relying on MeSH terms. After this, 509 articles were eliminated (17 abstracts of articles published in non-English languages, 353 duplicates, 71 in vitro or animal clinical studies, 39 were not pertinent, in 6 the Ethics Committee approval was not provided, and 23 were case reports and case series). A total of 25 articles were analyzed according to title and abstracts. Nine articles were excluded because impertinent, while the remaining fourteen were assessed for eligibility, included, and screened in this review. The review process is summarized in the flowchart shown in Figure 1.

Table S3 (Supplementary Materials) presents the studies that were excluded from this review along with the reasons for their exclusion [30,31,32,33,34,35,36,37,38,39,40]. The included studies were divided into two categories: controlled intervention studies [3,4,41,42,43,44,45] and observational cohort studies [46,47,48,49,50,51,52].

### Risk of Bias

The articles selected for this review were assessed for risk of bias using the ROBINS-I tool, as detailed in Table 1. The evaluation was carried out following the criteria outlined in the ROBINS-I assessment tool shown in Table S4 (Supplementary Materials). Table S5 details the bias risk of the studies included in this review as evaluated by the ROBINS-I tool. This review found a moderate risk of bias across the included studies.

Table 2 shows patient’s baseline data from the selected studies. Table S6 (Supplementary Materials) summarizes study details, including design, methods, results, and conclusions.

Table S7 (Supplementary Materials) presents the NHLBI Quality Assessment Tool for Observational Cohort and Cross-Sectional Studies.

## 4. Discussion

Anchorage has been a significant issue in orthodontics since the advent of permanent appliances and has garnered significant attention. Orthodontics has made use of a number of these extra-dental anchor types, including the traditional osteo-integrated implants, mini plates [53,54,55] and, more recently, mini-implants. The benefits of mini-implants are their great adaptability, ease of surgical implantation, and inexpensive cost [56,57,58]. The success rate for implant anchorage ranges between 85 and 95% [59,60,61] according to the literature; however, complications will inevitably develop despite its simplicity, in part due to the relatively inexperienced clinicians executing it. The aforementioned complications can be further subdivided into four categories: surgical complications, which occur during TADs placement, orthodontics complications, which occur after its mechanical loading, soft tissue issues, and removal complications [62].

### 4.1. Surgical Complications

#### Periodontal Ligament Injury, Root Contact with or Without Pulpal Involvement

Orthodontic miniscrews placed interradicularly run the danger of injuring the dental root or the periodontal ligament. Osteosclerosis, dentoalveolar ankylosis, and tooth vitality loss are possible side effects of root damage [63]. The prognosis of the tooth will probably not be affected by trauma to the external dental root if there is no pulpal involvement. After the orthodontic miniscrew is removed, the periodontium and the dental roots injured by the device are shown to fully heal in 12 to 18 weeks [64,65,66]. Appropriate radiological planning, which includes a surgical guide with panoramic and periapical radiographs, is required to determine the safest location for interradicular miniscrew placement. The maxillary buccal region’s thickest interradicular bone is located 5 to 8 mm from the alveolar crest, in the space between the second premolar and the first molar. The maximum amount of interradicular bone in the mandibular buccal region is located approximately 11 mm from the alveolar crest, either between the first and second molars or between the second premolar and the first molar [67,68,69]. It is common for the clinician to unintentionally draw the hand-driver toward their body during interradicular implantation in the posterior region, changing the angle of insertion and raising the possibility of root contact. The physician may be thinking about using a finger wrench or moving the hand-driver slightly away from the body with every motion to prevent this. With topical anesthetic, the patient will feel more sensation if the miniscrew starts to near the periodontal ligament. The miniscrew may halt or start to demand more insertion force if root contact is made. The physician should undo the miniscrew two or three turns and do a radiographic examination if trauma is suspected [70,71,72]. Another variable to consider as concerns root proximity is horizontal and vertical inclination. It has been shown that, in frontal view, vertical inclinations usually are approximately 50 degrees in the upper jaw and 60 degrees in the lower jaw. Mini-implants placed in the maxilla tend to be inserted at a more slanted angle compared to those in the mandible. This difference may result from the different perspectives when placing miniscrews in a reclining patient; in the maxilla, the drill’s direction is more visible when slanted, while, in the mandible, the drilling direction is observed from above along the dental arch. This difference in view influences the insertion angle of the miniscrews between the two jaws [43]. As concerns horizontal inclination in the axial view, the implant in the left mandible was placed nearly perpendicular to the bone surface, whereas implants in the left maxilla and right mandible were angled more distally. This variation is influenced by the sitting patient’s viewpoint, with drilling direction in the left mandible being easier to observe. Angling the implant opposite to the direction of the applied traction force may provide stronger anchorage due to increased stability [43]. As concerns dental pulp necrosis it has been shown that, among patients who exhibited a negative reaction to cold testing, TADS were often placed at the second rugae. It is reasonable to infer that inserting them more posteriorly towards the third rugae would better maintain vitality. In the anterior maxilla, proper insertion of the TAD is crucial to prevent damage to the incisor roots. Perpendicular insertion should be avoided and vertical placement to the bone surface is preferred to ensure the safety of this area [48,62]. Clinicians can place miniscrews either vertically or at an angle, as no insertion angle has been proven superior. The choice should depend on patient-specific factors such as anatomy, tooth and root position, and the type of force applied. This decision does not impact the clinical survival of miniscrews [3].

The results of vitality testing are, perhaps unsurprisingly, inconclusive. Vitality testing in cases of trauma is notoriously unreliable and this would seem to be borne out in this study [46]. The literature shows that proximity between the miniscrew and the root has moderate correlation for miniscrews’ failure [4].

### 4.2. Miniscrew Slipping

Miniscrew insertion with high forces can lead to its slippage. This can occur when the miniscew is inserted with a decreased insertion angle lower than 30° with respect to the occlusal plane. This complication can be prevented by starting the insertion at an obtuse angle to the occlusal plane and then reducing it to two to three turns while tightening the screw in and by applying light forces [61,72].

### 4.3. Nervous Damage

Another surgical complication that can occur is sensitive alteration, such as hypoesthesia. This could be due to indirect damage: nerve compression or direct nerve involvement. Specifically, an interesting area for this problem is the anterior maxilla. The course of the nasopalatine canal is still debated to date. The anatomical topography of the area has been already explored regarding fixture placement, and the same goes for TADs placement [64,73].

### 4.4. Subcutaneous Emphysema

It is a condition that can occur during different dental practices. It is most common during dental extraction or while using high-speed, air-driven surgical drills and compressed air syringes. In this instance, miniscrew placement can cause subcutaneous emphysema, which is air insufflation into the skin or submucosa that causes swelling. Only a few cases have been reported in the literature. The treatment consists of the RICE protocol application (Table 3) and a strict follow-up up to 10 days [74,75,76].

### 4.5. Sinus Perforation

During TADs placement several cases of sinus perforation have been documented. This can occur when the floor of the maxillary sinus is thin. However, sinus perforation does not necessarily lead to mini-implant failure or sinusitis. When the maxillary sinus floor thickness is less than 6.0 mm, the risk of sinus perforation with 8 mm miniscrews is significantly higher, with an odds ratio of 21.63 (*p* < 0.001) compared to cases where the thickness is 6.0 mm or more. Therefore, it is recommended to have at least 6.0 mm of sinus floor thickness to minimize the chance of maxillary sinus perforation during miniscrew insertion [65]. Penetration depth of the mini-implant causes changes in the sinus tissue conformation. Indeed, a thickening of 0.6 mm of the sinus membrane happens. A significantly higher value was observed around miniscrews that penetrated the sinus by more than 1 mm compared to those with less than 1 mm penetration (*p* < 0.017). This indicates that deeper penetration into the sinus is associated with greater effects than shallow penetration [50].

### 4.6. Bone Overheating

As concerns bone stress during TADs removal, a special note has to be made regarding temperature. The peak without irrigation can reach up to 50.9 degrees at 3 mm depth, while drilling with saline solution irrigation can decrease the heating to 37.4 degrees at 12 mm [64,77].

### 4.7. Orthodontic Complications: Failure of the Static Anchoring, Miniscrew Relocation

In the literature, there are only a few articles that discuss these variables. However, lateral forces on miniscrews may be unfavorable, but this needs more investigation. Minor deviations from the recommended insertion angle likely do not cause significant problems, making the technique suitable for less experienced practitioners, especially when deviations are necessary due to impacted teeth or clefts [50].

Orthodontic miniscrews can withstand forces effectively if there is sufficient bone support after a healing period. Clinically, loading is often delayed until around 3 months post-insertion, similar to traditional dental implants, as postponing the loading time reduces the risk of failure [45].

Both bone density and peri-implant soft tissue play a key role. Greater bone density reduces stress at the bone–screw interface and thin and keratinized mucosa, like the mid-palatal area, and stationary anchorage [22,78].

Under orthodontic loading, orthodontic miniscrews can stay rather stable, although not perfectly stationary. Orthodontic miniscrews achieve stability mainly through mechanical retention and can be shifted within the bone, while dental implants osteointegrate. Some cases have shown miniscrews tilting and extrusion of −1.0 to 1.5 mm when loaded with 400 g of force for 9 months. To accommodate this potential movement, clinicians are advised to maintain a safety margin of at least 2 mm between the miniscrew and any nearby anatomical structures to allow miniscrew migration [79].

### 4.8. Mechanical Complications

Miniscrew torsional stress, fracture, and bending: Excessive torsional forces can bend, fracture the miniscrew, or fracture the peri-implant bone, increasing the mobility of the screw [61,62,72]. These complications can be avoided either by using pilot drills in dense cortical [61,72] or by turning the screw two or three turns to reduce stress between screw and bone [72] and by removing the surrounding bone [60]. In case of screw fracture, it must be removed. If the TAD is subgingival, it is removed by making an incision; otherwise, no incision is needed to remove it if the TAD is supragingival [62].

### 4.9. Soft Tissue Complications

#### Scarring

Compared to insertion sites in the mucogingival junction or connected gingiva, TADs in the alveolar mucosa exhibited less scar development among the miniscrew-related parameters. According to histology, the alveolar mucosa is made up of far more elastic fibers than collagen, vascularized loose connective tissue, and nonkeratinized epithelium. These histological characteristics may help promote better wound healing. Maxillary buccal insertion sites have a higher likelihood of noticeable scarring than mandibular insertion sites. Miniscrews are more frequently placed in the attached gingiva of the maxilla than in the mandible because the attached gingiva in the maxillary buccal region is wider. Additionally, the flat gingival biotype typical of the maxillary buccal interdental area tends to be more susceptible to scarring [49].

Moreover, mini-screws placed between the second and third maxillary molars showed a significantly higher incidence than those placed between the first and second molars or between the first molar and second premolar [44].

Alternatively, the notable variations in scarring in the maxilla and mandible could have resulted from the disparities in bone composition between these two locations. Modifications in the food debris content, which are purportedly more common in the maxilla extraction sockets, could potentially impede the healing process following miniscrew extraction [49,80,81].

### 4.10. Aphthous Ulcerations

Small aphthous ulcerations can develop on the buccal mucosa next to the miniscrew head or around the miniscrew shaft. Minor aphthous ulcers are mainly caused by trauma to the soft tissues but can also result from bacterial infections, allergic reactions, hormonal changes, vitamin deficiencies, and genetic predispositions. Aphthae are defined as mildly painful ulcers that affect non-keratinized mucosa; these ulcers are self-limiting and heal without leaving scars in 7 to 10 days [82]. It is generally possible to prevent ulceration and improve patient comfort by using a wax pellet, a healing abutment, or a big elastic separator over the head of the miniscrew and by using chlorhexidine every day. Although it does not seem to be a direct risk factor for miniscrew stability, the development of an aphthous ulceration may indicate increased soft tissue inflammation [72].

### 4.11. Soft Tissue Overgrowth

When miniscrews are placed into the alveolar mucosa, especially in the jaw, soft tissue may conceal them. Within a day following placement, the head of the miniscrew and its attachments (like coil springs or elastic chains) may become covered by loose gum tissue that bunches up and rubs against them. This soft tissue growth can worry patients, who might think the miniscrew has fallen out, and it can also pose a risk to the stability of the miniscrew [83]. Tissue will probably cover any miniscrew attachments (coil springs and elastic chain) that are in contact with tissues. Light finger pressure can reveal the relatively thin, soft tissue covering the miniscrew, usually without the need for a cut or local anesthesia. The application of an elastic separator or healing abutment can reduce the amount of soft tissue overgrowth. Apart from its antibacterial characteristics, chlorhexidine also slows down the process of epithelization and can decrease the growth of soft tissues [84]. Overgrowth, defined as soft tissue partially or fully covering the miniscrew head, typically does not cause serious issues but can be a time-consuming nuisance. It may be prevented by reducing insertion depth or using implants with longer necks, with the latter preferred for ensuring better primary and long-term stability. Maintaining good oral hygiene is also important to avoid complications [52].

### 4.12. Peri-Implant Disease

Another complication that can affect TADs is peri-implant disease. Periodontal disease’s pathogenic mechanism results from bacteria and the chemicals they generate in plaque. Bacterial antigens and their toxic substances, such as toxins and enzymes, can damage periodontal tissues and provoke an immune response from the host. Peri-implanititis and periodontitis are similar in pathogenic microorganisms; however, peri-implanititis has a wider spectrum of bacteria and a stronger correlation with the red and orange complex [85]. From a microbiological point of view, failed TADs showed higher presence of microorganisms linked to periodontal disease, including Prevotella nigrescenis, Filifactor alocis, Porphyromonas gingivalis, and Fusobacterium nucleatum [86]. The literature suggests that the prevalence of inflammation (10.2%) between the palatal region and buccal fold can be overlapped. Peri-implant flogosis can be treated with an iodine-containing solution and chlorhexidine gel [47]. At longer follow-up times, up to 10 years after placement, the anterior alveolar region of the maxilla presented chronic inflammation mostly due to miniscrew placement in that area [51].

### 4.13. Removal Complication

#### Bone Damage

During TADs explantation, a phenomenon that can be experienced is bone sequester that can be addressed to undiagnosed diseases related to bone metabolism or smoking. Indeed, during the explantation procedure, excessive forces can lead to bone compression, thus necrosis, or insufficient irrigation [64].

In some cases, TADs can undergo partial osteointegration thanks to mechanical retention, thus leading to hard removal. The miniscrew usually can be extracted some days after the first attempt of removal [66].

Table 4 shows a summary of TAD’s complication incidence on the basis of the included articles for this review.

## 5. Conclusions

- Miniscrews offer flexible and cost-effective orthodontic anchorage, yet their use requires careful planning and execution to avoid complications.
- Surgical precision, guided by radiological assessment, is crucial to prevent periodontal ligament injury and root contact.
- While orthodontic and mechanical issues can arise, diligent monitoring and post-placement care can mitigate these risks.
- Soft tissue complications, such as scarring and ulcerations, underscore the importance of meticulous patient management. Similarly, the risk of peri-implant disease and bone damage during removal highlights the need for thorough assessment and intervention.
- Despite challenges, advancements continue to improve miniscrew anchorage efficacy. With proper planning and ongoing monitoring, miniscrews can significantly enhance orthodontic outcomes, ensuring patient satisfaction and treatment success.

## Figures and Tables

**Table 1:** Rrisk of bias in the included studies, with green symbols indicating low risk and yellow indicating high risk.

References (Authors, Year of Publication)	Random Sequence Generation	Allocation Concealment	Blinding	Incomplete Outcome Data	Selective Reporting
Golshah A et al., 2021 [3]	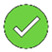	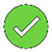	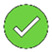	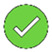	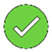
Aboshady H. et al., 2022 [4]	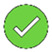	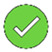	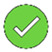	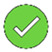	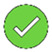
Fäh et al., 2014 [41]	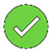	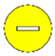	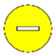	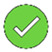	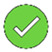
Motoyoshi et al., 2015 [42]	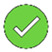	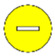	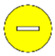	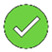	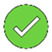
Shinohara et al., 2013 [43]	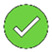	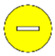	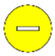	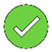	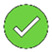
Wang et al., 2010 [44]	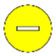	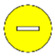	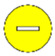	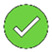	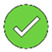
Xin et al., 2022 [45]	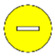	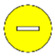	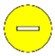	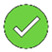	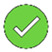

**Table 2:** Patient baseline data from the selected studies.

References (Authors, Year of Publication and Study Design)	N° of Patients and % Women	Mean Age (Years), Mean (SD or Range)	Inclusion and Exclusion Criteria	Type of Complication
Golshah A et al., 2021, RCT [3]	25 W: 64%		Inclusion: Skeletal class II division 1 malocclusion with full cusp class II molar relationship and a normal maxillary angle relative to the mandibular plane (25° ± 5°), required extraction of the first premolars and maxillary canine retraction assisted by miniscrew insertion in the maxillary buccal plate. Exclusion: Missing or impacted teeth at the treatment site, periodontal disease or bone loss, insufficient attached gingiva, nonoptimal frenum position, medication use, facial asymmetry, cleft palate/lip, craniofacial disorders, syndromes, cigarette smoking, and poor oral hygiene.	pain
Aboshady H. et al., 2022, RCT [4]	44 W: N.R.	age (22.56 ± 3.47 years) range 18–30	Inclusion: Maxillary first premolars’ extraction, biprotrusion and Class II division 1 with maximum anchorage Nonsmoker No maxillary partial except for 3rd molars Exclusion: smokers, bleeding disorder bone disorder, osteoporosis on anticoagulant therapy, on medication, cleft lip and palate.	Moderate correlation between root proximity and miniscrew failure rate
Fabbroni G. et al., 2004, prospective study [46]	55 W: 1.81%	Range 16–52 yo	Inclusion: Patients who had mandibular fractures and required control of their occlusion using transalveolar screws were included in the study. Exclusion: N.R.	Small granulation area in the screw hole. Root contact.
Fäh et al., 2014, retrospective study [41]	146 W: 72.6%	Mean age 21 yo	Inclusion: patients treated with palatal implant anchorage between 1999 and 2010. Surgeon with >10 miniscrew insertion Patients’ available data: age, gender, type of implant, surgical procedure, placement location. Good general health Exclusion: N.R.	Loss of primary stability, prolonged pain. Secondary bleeding, perforation of nasal floor, necrotic mucosa, sensory impairment, disturbed wound healing, fracture of the implant.
Gurdan et al., 2018, retrospective study [47]	47 patients W: 74.4%	Mean age 20–30 yo	Inclusion: orthodontic treatment with self-drilling miniscrews between 11/2014 and 11/2016. Exclusion: N.R.	Soft tissue infection, screw mobility
Hourfar et al., 2017, retrospective study [48]	284 patients W: 64%	14.4 years old ± 8.8 years old.	Inclusion: orthodontic fixed therapy with miniscrew. Unrestored maxillary front teeth without previous trauma or dental procedures. Exclusion: generalized diseases, craniofacial malformation, chemo or radiotherapy during tooth development, craniofacial trauma, history of endotracheal intubation, dental malformation, severe upper anterior crowding, periodontal disease, previous orthodontic treatment, tooth loss or agenesis (except for 3rd molars), previous stripping or occlusal adjustment of the maxillary anterior teeth, no medications like tranquilizers, sedatives or analgesics	No response to pulp sensibility and no tooth vitality. Root perforations
Jung et al., 2014, cohort study [49]	66 patients W: 78.78%	Mean age 28.58 years old	Inclusion: patients that went through orthodontic therapy with miniscrews removed at least 1 year (12 to 58 months range) prior to the assessment. Presence of soft tissue scarring at miniscrew removal sites. Exclusion: N.R.	N.R. (not reported)
Jia et al., 2018, retrospective study. [50]	32 patients W: 68.75%	Mean age of 28 ± 6 yo	Inclusion: Chinese patients that went through orthodontic fixed therapy for distalization of the upper dental arch with mini implants inserted in the infrazygomatic crest as anchorage. With a CBCT right before miniscrew removal and an image with the clear and complete sinus floor. Exclusion: miniscrews with tooth root contact.	Mobility
Motoyoshi et al., 2015 prospective study [42]	45 patients W: 58.3%	age 23.3 ± 8.9 years	Inclusion: miniscrews placed in the buccal alveolar bone between the upper second premolar and upper first molar at Nihon University Dental Hospital. Exclusion: N.R.	N.R.
Shinohara et al., 2013 prospective study [43]	50 patients W: 70%	21.8 ± 5.7 years;	Inclusion: miniscrews in the buccal alveolar bone between second premolar and first molar used as orthodontic anchorage. Exclusion: cases of 2nd premolar extractions or miniscrew penetrated the maxillary sinus.	N.R.
Takaki et al., 2010 Retrospective study [51]	455 patients W: 78.6%	Mean age 25.7 ± 9.8 years	Inclusion: patients having malocclusion, impaction, syndromes, cleft palate/lip, jaw deformity, that required orthodontic therapy. All of them underwent surgery between 11/2000 and 06/2009 at the Tokyo dental college Chiba Hospital. Exclusion: N.R.	Inflammation of soft tissue around the miniscrew
Ziebura et al., 2012 Retrospective study [52]	51 W: 43%	mean age 15.1 years, standard deviation 4.9.	Inclusion: Jet screws placed in the palatal slope between 12/2009 and 11/2011. Exclusion: patients who aborted treatment and patients who did not agree with using their clinical photographs for scientific use.	Implant loss, lessening, mild bleeding, overgrowth of soft tissue onto the implant head.
Wang et al., 2010 retrospective study [44]	54 W: 62.9%	Age from 16 to 32 years, average of 21.8 years.	Inclusion: patients with the following data were included: basic information, angle of screw placement, and information about the buccal lesions caused by the corresponding miniscrew. Exclusion: N.R.	Buccal trauma, overgrowth of soft tissue onto the screw’s head.
Xin et al., 2022 Retrospective study [45]	347 patients W: 84.1%	Mean Age 25.62 ± 7.43 Years	Inclusion: patients who underwent orthodontic treatment with titanium alloy miniscrews as orthodontic anchorage between 01/2017 and 12/2020. Exclusion: pathologic bone loss, lesions or bone disorders or medication affecting bone density, cysts.	N.R.

**Table 3:** RICE protocol.

Rest	Avoid harsh chewing
Ice	Apply ice packs on the affected area intermittently for 20 min on and off during the first day. Ice has analgesic effects.
Compression	Compression with ice packages reduces swelling.
Elevation	Lie down, while maintaining the interested zone elevated.

**Table 4:** Summary of TADs complication incidence.

Study	Complications/Incidence (%)	Quantitative Indicators	Identified Risk Factors
Golshah et al., 2021 [3]	Failure: 24% (90°) vs. 12% (45°)	PTV 1 mo: 4.07 ± 7.76 (90°), 3.72 ± 9.19 (45°).	Insertion angle not significant; bone density may influence stability.
Aboshady et al., 2022 [4]	Failure: 7.14% (guide) vs. 16.6% (free-hand)	—	Root proximity = moderate predictor of failure (free-hand).
Fabbroni et al., 2004 [46]	Root contact: 27.1% (11.2% major, 15.9% minor). Non-vital teeth: 17 cases	—	Greater contact severity = more pulp effects.
Fäh et al., 2014 [41]	Complications: 24%. Primary stability loss 6.7%. Pain 6.7%. Bleeding 5.8%. Necrosis 1.9%. Nasal perforation 1.9%. Fracture 2.3%.	—	Low stability, flap approach, bone quantity, surgeon experience.
Gurdan et al., 2018 [47]	Soft-tissue infections 6.3–33.3%. Mobility 3.1–20.8%. No root injury.	Success 89.8%. Loading 8.1 ± 3.3 mo.	Buccal fold ↑ mobility (*p* = 0.034). Intrusion ↑ mobility (*p* = 0.036). Age/gender NS.
Hourfar et al., 2017 [48]	PST loss: 1.06% patients; 0.53% per OMI; 0.18% per tooth. No root injury on radiographs.	568 OMIs.	Position at R-2 ruga significant (*p* = 0.008). Other factors NS.
Jung et al., 2015 [49]	Scarring 44.6%. TAD success 92.1%.	—	Flat biotype ↑ scarring RR = 2.5. Maxillary buccal sites RR = 2.1 vs. mandibular. Attached gingiva RR = 2.5 vs. alveolar mucosa.
Jia et al., 2018 [50]	Sinus penetration: 78.3%. Success: 96.7%. Membrane thickening: 88.2% (>1 mm penetration).	Membrane thickness 1.0 vs. 0.2 mm (*p* < 0.05).	Penetration > 1 mm ↑ membrane changes. Recommend ≤ 1.0 mm.
Motoyoshi et al., 2015 [42]	Sinus perforation: 9.8%. Failures: 1/8 perforated vs. 4/74 non (NS).	Penetration depth 0.79 ± 0.39 mm. SFT: 5.6 (perforated) vs. 10.5 mm (non) (*p* = 0.000).	SFT < 6 mm OR = 21.6 for perforation (*p* < 0.001).
Shinohara et al., 2013 [43]	Root contact ≈ 20%.	Angles: Maxilla 48–50°, Mandible 57–63°.	Buccal maxillary right region ↑ distal root contact (significant).
Takaki et al., 2010 [51]	Overall failure: 6.9%. By type: Micro-screws 7%, mini-screws 6%, palatal screws 11%, plates 6%.	904 TADs. Age 25.7 ± 9.8 yrs.	Younger age ↑ palatal failure (up to 36–50%). Mandibular-body plates = 15% failure.
Ziebura et al., 2012 [52]	Permanent failure: 3%. Loosening: 3%. Gingival overgrowth: 12.1%.	—	Lateral loading → loosening. Short 3 mm neck ↑ overgrowth. Patient manipulation caused 1 failure.
Wang et al., 2010 [44]	Buccal mucosal lesions: 11.8%.	Incidence by angle: 10–30° → 28.1%; 30–60° → 8.6%; 60–80° → 4.4%.	Site and vertical position significant (*p* = 0.00). Low insertion angle ↑ lesions.
Xin et al., 2022 [45]	Failures: 17.1% failed once; 5.29% failed ≥2. Never failed 77.6%.	—	Younger age ↑ failure. Early loading ↑ failure. Retromaxillary/retromandibular sites ↓ stability. Removable appliances ↑ failures. More screws/patient ↑ failures.

## Data Availability

The raw data supporting the conclusions of this article will be made available by the authors on request.

## Supplementary Materials

The following supporting information can be downloaded at: https://www.mdpi.com/article/10.3390/dj13120582/s1, Table S1: PRISMA-ScR checklist; Table S2: Search strategies for electronic databases; Table S3: Summary table of studies excluded in this systematic review; Table S4: Criteria to assess bias risk using the ROBINS-I tool; Table S5: Risk of bias of the included studies through ROBINS-I assessment tool; Table S6: Evidence of the included studies; Table S7: NHLBI Quality Assessment Tool for Observational Cohort and Cross-Sectional Studies.
