# Supplementary material for: Clinical Management of Orthodontic Miniscrew Complications: A Scoping Review
Source: Dent J (Basel). 2025 Dec 5;13(12):582. doi: 10.3390/dj13120582 (PMC12732202; doi:10.3390/dj13120582)
Supplement: Supplementary file 1 [file dentistry-13-00582-s001.zip › dentistry-3973989-supplementary.pdf]

Table S1. (PRISMA-ScR) Checklist.

SECTION	ITEM	PRISMA-ScR CHECKLIST ITEM	REPORTED ON PAGE #
**TITLE**			
Title	1	Identify the report as a scoping review.	Title
**ABSTRACT**			
Structured summary	2	Provide a structured summary that includes (as applicable): background, objectives, eligibility criteria, sources of evidence, charting methods, results, and conclusions that relate to the review questions and objectives.	Abstract
**INTRODUCTION**			
Rationale	3	Describe the rationale for the review in the context of what is already known. Explain why the review questions/objectives lend themselves to a scoping review approach.	Initial introduction
Objectives	4	Provide an explicit statement of the questions and objectives being addressed with reference to their key elements (e.g., population or participants, concepts, and context) or other relevant key elements used to conceptualize the review questions and/or objectives.	End of introduction
**METHODS**			
Protocol and registration	5	Indicate whether a review protocol exists; state if and where it can be accessed (e.g., a Web address); and if available, provide registration information, including the registration number.	Dedicated section in M&M
Eligibility criteria	6	Specify characteristics of the sources of evidence used as eligibility criteria (e.g., years considered, language, and publication status), and provide a rationale.	Dedicated section in M&M
Information sources*	7	Describe all information sources in the search (e.g., databases with dates of coverage and contact with authors to identify additional sources), as well as the date the most recent search was executed.	Dedicated section in M&M

SECTION	ITEM	PRISMA-ScR CHECKLIST ITEM	REPORTED ON PAGE #
Search	8	Present the full electronic search strategy for at least 1 database, including any limits used, such that it could be repeated.	Dedicated section in M&M
Selection of sources of evidence†	9	State the process for selecting sources of evidence (i.e., screening and eligibility) included in the scoping review.	Dedicated section in M&M
Data charting process‡	10	Describe the methods of charting data from the included sources of evidence (e.g., calibrated forms or forms that have been tested by the team before their use, and whether data charting was done independently or in duplicate) and any processes for obtaining and confirming data from investigators.	Dedicated section in M&M
Data items	11	List and define all variables for which data were sought and any assumptions and simplifications made.	Dedicated section in M&M
Critical appraisal of individual sources of evidence§	12	If done, provide a rationale for conducting a critical appraisal of included sources of evidence; describe the methods used and how this information was used in any data synthesis (if appropriate).	Dedicated section in M&M
Synthesis of results	13	Describe the methods of handling and summarizing the data that were charted.	Dedicated section in M&M
**RESULTS**			
Selection of sources of evidence	14	Give numbers of sources of evidence screened, assessed for eligibility, and included in the review, with reasons for exclusions at each stage, ideally using a flow diagram.	Dedicated table
Characteristics of sources of evidence	15	For each source of evidence, present characteristics for which data were charted and provide the citations.	Dedicated table

SECTION	ITEM	PRISMA-ScR CHECKLIST ITEM	REPORTED ON PAGE #
Critical appraisal within sources of evidence	16	If done, present data on critical appraisal of included sources of evidence (see item 12).	Dedicated table
Results of individual sources of evidence	17	For each included source of evidence, present the relevant data that were charted that relate to the review questions and objectives.	Dedicated table
Synthesis of results	18	Summarize and/or present the charting results as they relate to the review questions and objectives.	Dedicated table
**DISCUSSION**			
Summary of evidence	19	Summarize the main results (including an overview of concepts, themes, and types of evidence available), link to the review questions and objectives, and consider the relevance to key groups.	Followed
Limitations	20	Discuss the limitations of the scoping review process.	Followed
Conclusions	21	Provide a general interpretation of the results with respect to the review questions and objectives, as well as potential implications and/or next steps.	Followed
**FUNDING**			
Funding	22	Describe sources of funding for the included sources of evidence, as well as sources of funding for the scoping review. Describe the role of the funders of the scoping review.	None

Table S2. Search strategies for electronic databases.

Database	Search strategy
PubMed (MEDLINE)	#1 "Orthodontic Anchorage Procedures" [MESH] OR (Anchorage Procedure, Orthodontic) OR (Anchorage Procedures, Orthodontic) OR (Orthodontic Anchorage Procedure) OR (Procedure, Orthodontic Anchorage) OR (Orthodontic Anchorage Techniques) OR (Anchorage Techniques, Orthodontic) OR (Orthodontic Anchorage Technique) OR (Techniques, Orthodontic Anchorage)<br><br>#2 "Dental Implants " [MESH] OR (Implant, Dental) OR (Implants, Dental) OR (Dental Implants, Mini) OR (Dental Implant, Mini) OR (Mini Dental Implant) OR (Mini Dental Implants) OR (Dental Prostheses, Surgical) OR (Surgical Dental Prostheses) OR (Surgical Dental Prosthesis) OR (Prosthesis, Surgical Dental)<br><br>#3 "Intraoperative Complications" [MESH] OR (Complication, Peroperative) OR (Complications, Peroperative) OR (Peroperative Complication) OR (Complication, Intraoperative) OR (Intraoperative Complication) OR (Injuries, Surgical) OR (Surgical Injuries)<br><br>#4 "Root Resorption" [MESH] OR (Resorption, Root) OR (Resorptions, Root) OR (Root Resorptions)<br><br>#5 "Cicatrix" [MESH] OR (Scar) OR (Scars) OR (Cicatrization) OR (Scarring)<br><br>#6 "Mouth Mucosa" [MESH] OR (Mucosa, Mouth) OR (Oral Mucosa) OR (Mucosa, Oral) OR (Buccal Mucosa)<br><br>#7 #1 AND #2 AND #3<br><br>#8 #1 AND #2 AND #4<br><br>#9 #1 AND #2 AND #5 AND #6

SCOPUS	#1 “Orthodontic Anchorage Procedures” [MESH] OR (Anchorage Procedure, Orthodontic) OR (Anchorage Procedures, Orthodontic) OR (Orthodontic Anchorage Procedure) OR (Procedure, Orthodontic Anchorage) OR (Orthodontic Anchorage Techniques) OR (Anchorage Techniques, Orthodontic) OR (Orthodontic Anchorage Technique) OR (Techniques, Orthodontic Anchorage)<br><br>#2 “Dental Implants ” [MESH] OR (Implant, Dental) OR (Implants, Dental) OR (Dental Implants, Mini) OR (Dental Implant, Mini) OR (Mini Dental Implant) OR (Mini Dental Implants) OR (Dental Prostheses, Surgical) OR (Surgical Dental Prostheses) OR (Surgical Dental Prosthesis) OR (Prosthesis, Surgical Dental)<br><br>#3 “Intraoperative Complications” [MESH] OR (Complication, Peroperative) OR (Complications, Peroperative) OR (Peroperative Complication) OR (Complication, Intraoperative) OR (Intraoperative Complication) OR (Injuries, Surgical) OR (Surgical Injuries)<br><br>#4 “Root Resorption” [MESH] OR (Resorption, Root) OR (Resorptions, Root) OR (Root Resorptions)<br><br>#5 “Cicatrix” [MESH] OR (Scar) OR (Scars) OR (Cicatrization) OR (Scarring)<br><br>#6 “Mouth Mucosa” [MESH] OR (Mucosa, Mouth) OR (Oral Mucosa) OR (Mucosa, Oral) OR (Buccal Mucosa)<br><br>#7 #1 AND #2 AND #3<br><br>#8 #1 AND #2 AND #4<br><br>#9 #1 AND #2 AND #5 AND #6
Web of Sciences	#1 “Orthodontic Anchorage Procedures” [MESH] OR (Anchorage Procedure, Orthodontic) OR (Anchorage Procedures, Orthodontic) OR (Orthodontic Anchorage Procedure) OR (Procedure, Orthodontic Anchorage) OR (Orthodontic Anchorage Techniques) OR (Anchorage

	Techniques, Orthodontic) OR (Orthodontic Anchorage Technique) OR (Techniques, Orthodontic Anchorage)<br><br>#2 “Dental Implants ” [MESH] OR (Implant, Dental) OR (Implants, Dental) OR (Dental Implants, Mini) OR (Dental Implant, Mini) OR (Mini Dental Implant) OR (Mini Dental Implants) OR (Dental Prostheses, Surgical) OR (Surgical Dental Prostheses) OR (Surgical Dental Prosthesis) OR (Prosthesis, Surgical Dental)<br><br>#3 “Intraoperative Complications” [MESH] OR (Complication, Peroperative) OR (Complications, Peroperative) OR (Peroperative Complication) OR (Complication, Intraoperative) OR (Intraoperative Complication) OR (Injuries, Surgical) OR (Surgical Injuries)<br><br>#4 “Root Resorption” [MESH] OR (Resorption, Root) OR (Resorptions, Root) OR (Root Resorptions)<br><br>#5 “Cicatrix” [MESH] OR (Scar) OR (Scars) OR (Cicatrization) OR (Scarring)<br><br>#6 “Mouth Mucosa” [MESH] OR (Mucosa, Mouth) OR (Oral Mucosa) OR (Mucosa, Oral) OR (Buccal Mucosa)<br><br>#7 #1 AND #2 AND #3<br><br>#8 #1 AND #2 AND #4<br><br>#9 #1 AND #2 AND #5 AND #6

Table S3. Summary table of studies excluded in this systematic review.

Excluded Studies	Exclusion Reasons
Lo Giudice et al., 2021 [1]	Systematic Review
Papageorgiou et al., 2012 [2]	Meta-analysis
Mohamed et al., 2018 [3]	Systematic Review
Inchingolo et al., 2023 [4]	Narrative Review
Ramirez-Ossa et al., 2020 [5]	Narrative Review
Kakali et al., 2019 [6]	Systematic Review
Yi et al., 2017 [7]	Systematic Review and Meta-Analysis
Tsui et al., 2012 [8]	Systematic Review

Mousa et al., 2023 [9]	Systematic Review
Khlef et al., 2018 [10]	Systematic Review and Meta-Analysis
Gintautaitè et al., 2018 [11]	Systematic Review

Table S4. Criteria for judging risk of bias in ROBINS-I assessment tool [12].

**1. Reaching risk of bias judgements for bias due to confounding**	
Low risk of bias (the study is comparable to a well-performed randomized trial with regard to this domain)	No confounding expected.
Moderate risk of bias (the study is sound for a non-randomized study with regard to this domain but cannot be considered comparable to a well-performed randomized trial)	(i) Confounding expected, all known important confounding domains appropriately measured and controlled for; and (ii) Reliability and validity of measurement of important domains were sufficient, such that we do not expect serious residual confounding.
Serious risk of bias (the study has some important problems)	(i) At least one known important domain was not appropriately measured, or not controlled for; or (ii) Reliability or validity of measurement of an important domain was low enough that we expect serious residual confounding.
Critical risk of bias (the study is too problematic to provide any useful evidence on the effects of intervention)	(i) Confounding inherently not controllable or (ii) The use of negative controls strongly suggests unmeasured confounding.
No information on which to base a judgement about risk of bias for this domain	No information on whether confounding might be present.
**2. Reaching risk of bias judgements for bias in selection of participants into the study**	
Low risk of bias (the study is comparable to a well-performed randomized trial with regard to this domain)	(i) All participants who would have been eligible for the target trial were included in the study; and (ii) For each participant, start of follow up and start of intervention coincided.

Moderate risk of bias (the study is sound for a non-randomized study with regard to this domain but cannot be considered comparable to a well-performed randomized trial)	(i) Selection into the study may have been related to intervention and outcome; and the authors used appropriate methods to adjust for the selection bias; or (ii) Start of follow-up and start of intervention do not coincide for all participants; and (a) the proportion of participants for which this was the case was too low to induce important bias; or (b) the authors used appropriate methods to adjust for the selection bias; or (c) the review authors are confident that the rate (hazard) ratio for the effect of intervention remains constant over time.
Serious risk of bias (the study has some important problems)	(i) Selection into the study was related (but not very strongly) to intervention and outcome; and This could not be adjusted for in analyses; or (ii) Start of follow up and start of intervention do not coincide; and A potentially important amount of follow-up time is missing from analyses; and The rate ratio is not constant over time.
Critical risk of bias (the study is too problematic to provide any useful evidence on the effects of intervention)	(i) Selection into the study was very strongly related to intervention and outcome; and This could not be adjusted for in analyses; or (ii) A substantial amount of follow-up time is likely to be missing from analyses; and The rate ratio is not constant over time.
No information on which to base a judgement about risk of bias for this domain	No information is reported about selection of participants into the study or whether start of follow up and start of intervention coincide.
**3. Reaching risk of bias judgements for bias in classification of interventions**	

Low risk of bias (the study is comparable to a well-performed randomized trial with regard to this domain)	(i) Intervention status is well defined; and (ii) Intervention definition is based solely on information collected at the time of intervention.
Moderate risk of bias (the study is sound for a non-randomized study with regard to this domain but cannot be considered comparable to a well-performed randomized trial)	(i) Intervention status is well defined; and (ii) Some aspects of the assignments of intervention status were determined retrospectively.
Serious risk of bias (the study has some important problems)	(i) Intervention status is not well defined; or (ii) Major aspects of the assignments of intervention status were determined in a way that could have been affected by knowledge of the outcome.
Critical risk of bias (the study is too problematic to provide any useful evidence on the effects of intervention)	(Unusual) An extremely high amount of misclassification of intervention status, e.g. because of unusually strong recall biases.
No information on which to base a judgement about risk of bias for this domain	No definition of intervention or no explanation of the source of information about intervention status is reported.
**4. Reaching risk of bias judgements for bias due to deviations from intended interventions**	
Low risk of bias (the study is comparable to a well-performed randomized trial with regard to this domain)	*Effect of assignment to intervention:* (i) Any deviations from intended intervention reflected usual practice; or (ii) Any deviations from usual practice were unlikely to impact on the outcome.<br><br>*Effect of starting and adhering to intervention:* The important co-interventions were balanced across intervention groups, and there were no deviations from

	the intended interventions (in terms of implementation or adherence) that were likely to impact on the outcome.
Moderate risk of bias (the study is sound for a non-randomized study with regard to this domain but cannot be considered comparable to a well-performed randomized trial)	*Effect of assignment to intervention:* There were deviations from usual practice, but their impact on the outcome is expected to be slight.<br><br>*Effect of starting and adhering to intervention:* (i) There were deviations from intended intervention, but their impact on the outcome is expected to be slight or (ii) The important co-interventions were not balanced across intervention groups, or there were deviations from the intended interventions (in terms of implementation and/or adherence) that were likely to impact on the outcome; and The analysis was appropriate to estimate the effect of starting and adhering to intervention, allowing for deviations (in terms of implementation, adherence and co-intervention) that were likely to impact on the outcome.
Serious risk of bias (the study has some important problems)	*Effect of assignment to intervention:* There were deviations from usual practice that were unbalanced between the intervention groups and likely to have affected the outcome.<br><br>*Effect of starting and adhering to intervention:* (i) The important co-interventions were not balanced across intervention groups, or there were deviations from the intended interventions (in terms of implementation and/or adherence) that were likely to impact on the outcome; and (ii) The analysis was not appropriate to estimate the effect of starting and adhering to intervention, allowing for

	deviations (in terms of implementation, adherence and co-intervention) that were likely to impact on the outcome.
Critical risk of bias (the study is too problematic to provide any useful evidence on the effects of intervention)	*Effect of assignment to intervention:* There were substantial deviations from usual practice that were unbalanced between the intervention groups and likely to have affected the outcome.<br><br>*Effect of starting and adhering to intervention:* (i) There were substantial imbalances in important co-interventions across intervention groups, or there were substantial deviations from the intended interventions (in terms of implementation and/or adherence) that were likely to impact on the outcome; and (ii) The analysis was not appropriate to estimate the effect of starting and adhering to intervention, allowing for deviations (in terms of implementation, adherence and co-intervention) that were likely to impact on the outcome.
No information on which to base a judgement about risk of bias for this domain	No information is reported on whether there is deviation from the intended intervention.
**5. Reaching risk of bias judgements for bias due to missing data**	
Low risk of bias (the study is comparable to a well-performed randomized trial with regard to this domain)	(i) Data were reasonably complete; or (ii) Proportions of and reasons for missing participants were similar across intervention groups; or (iii) The analysis addressed missing data and is likely to have removed any risk of bias.

Moderate risk of bias (the study is sound for a non-randomized study with regard to this domain but cannot be considered comparable to a well-performed randomized trial)	(i) Proportions of and reasons for missing participants differ slightly across intervention groups; and (ii) The analysis is unlikely to have removed the risk of bias arising from the missing data.
Serious risk of bias (the study has some important problems)	(i) Proportions of missing participants differ substantially across interventions; or Reasons for missingness differ substantially across interventions; and (ii) The analysis is unlikely to have removed the risk of bias arising from the missing data; or Missing data were addressed inappropriately in the analysis; or The nature of the missing data means that the risk of bias cannot be removed through appropriate analysis.
Critical risk of bias (the study is too problematic to provide any useful evidence on the effects of intervention)	(i) (Unusual) There were critical differences between interventions in participants with missing data; and (ii) Missing data were not, or could not, be addressed through appropriate analysis.
No information on which to base a judgement about risk of bias for this domain	No information is reported about missing data or the potential for data to be missing.
**6. Reaching risk of bias judgements for bias in measurement of outcomes**	
Low risk of bias (the study is comparable to a well-performed randomized trial with regard to this domain)	(i) The methods of outcome assessment were comparable across intervention groups; and (ii) The outcome measure was unlikely to be influenced by knowledge of the intervention received by study participants (i.e. is objective) or the outcome assessors were unaware of the intervention received by study

	participants; and (iii) Any error in measuring the outcome is unrelated to intervention status.
Moderate risk of bias (the study is sound for a non-randomized study with regard to this domain but cannot be considered comparable to a well-performed randomized trial)	(i) The methods of outcome assessment were comparable across intervention groups; and (ii) The outcome measure is only minimally influenced by knowledge of the intervention received by study participants; and (iii) Any error in measuring the outcome is only minimally related to intervention status.
Serious risk of bias (the study has some important problems)	(i) The methods of outcome assessment were not comparable across intervention groups; or (ii) The outcome measure was subjective (i.e. vulnerable to influence by knowledge of the intervention received by study participants); and The outcome was assessed by assessors aware of the intervention received by study participants; or (iii) Error in measuring the outcome was related to intervention status.
Critical risk of bias (the study is too problematic to provide any useful evidence on the effects of intervention)	The methods of outcome assessment were so different that they cannot reasonably be compared across intervention groups.
No information on which to base a judgement about risk of bias for this domain	No information is reported about the methods of outcome assessment.
**7. Reaching risk of bias judgements for bias in selection of the reported result**	
Low risk of bias (the study is comparable to a well-performed randomized trial with regard to this domain)	There is clear evidence (usually through examination of a pre-registered protocol or statistical analysis plan) that all reported results correspond to all intended outcomes, analyses and sub-cohorts.

Moderate risk of bias (the study is sound for a non-randomized study with regard to this domain but cannot be considered comparable to a well-performed randomized trial)	(i) The outcome measurements and analyses are consistent with an a priori plan; or are clearly defined and both internally and externally consistent; and (ii) There is no indication of selection of the reported analysis from among multiple analyses; and (iii) There is no indication of selection of the cohort or subgroups for analysis and reporting on the basis of the results.
Serious risk of bias (the study has some important problems)	(i) Outcomes are defined in different ways in the methods and results sections, or in different publications of the study; or (ii) There is a high risk of selective reporting from among multiple analyses; or (iii) The cohort or subgroup is selected from a larger study for analysis and appears to be reported on the basis of the results.
Critical risk of bias (the study is too problematic to provide any useful evidence on the effects of intervention)	(i) There is evidence or strong suspicion of selective reporting of results; and (ii) The unreported results are likely to be substantially different from the reported results.
No information on which to base a judgement about risk of bias for this domain.	There is too little information to make a judgement (for example if only an abstract is available for the study).

Table S5. Risk of bias of the studies included in this review through ROBINS-I assessment tool.

**Authors and Year of Publication**	**Signalling questions**	**Description**	**Response options**
Fabbroni G. et al 2004<br><br>[13]	**1. Bias due to confounding**		
	1.1 Is there potential for confounding of the effect of intervention in this study?<br><br>**If N/PN to 1.1:** the study can be considered to be at low risk of bias due to confounding and no further signalling questions need be considered		Y / PY / PN / N
	**If Y/PY to 1.1**: determine whether there is a need to assess time-varying confounding:		
	1.2. Was the analysis based on splitting participants' follow up time according to intervention received?<br><br>**If N/PN**, answer questions relating to baseline confounding (1.4 to 1.6)<br><br>**If Y/PY**, go to question 1.3.<br><br>1.3. Were intervention discontinuations or switches likely to be related to factors that are prognostic for the outcome?<br><br>**If N/PN**, answer questions relating to baseline confounding (1.4 to 1.6)<br><br>**If Y/PY**, answer questions relating to both baseline and time-varying confounding (1.7 and 1.8)		NA / Y / PY / PN / N / NI<br><br>NA / Y / PY / PN / N / NI
	Questions relating to baseline confounding only		
	1.4. Did the authors use an appropriate analysis method that controlled for all the important confounding domains?		NA / Y / PY / PN / N / NI

	1.5. **If Y/PY to 1.4**: Were confounding domains that were controlled for measured validly and reliably by the variables available in this study?		NA / Y / PY / PN / N / NI
	1.6. Did the authors control for any post-intervention variables that could have been affected by the intervention?		NA / Y / PY / PN / N / NI
	Questions relating to baseline and time-varying confounding		
	1.7. Did the authors use an appropriate analysis method that controlled for all the important confounding domains and for time-varying confounding?		NA / Y / PY / PN / N / NI
	1.8. **If Y/PY to 1.7**: Were confounding domains that were controlled for measured validly and reliably by the variables available in this study?		NA / Y / PY / PN / N / NI
	Risk of bias judgement		**Low** / Moderate / Serious / Critical / NI
	Optional: What is the predicted direction of bias due to confounding?		Favours experimental / Favours comparator / Unpredictable
	**2. Bias in selection of participants into the study**		
	2.1. Was selection of participants into the study (or into the analysis) based on participant characteristics observed after the start of intervention?<br>**If N/PN to 2.1:** go to 2.4		Y / PY / PN / N / NI

	2.2. **If Y/PY to 2.1**: Were the post-intervention variables that influenced selection likely to be associated with intervention?<br><br>2.3 **If Y/PY to 2.2**: Were the post-intervention variables that influenced selection likely to be influenced by the outcome or a cause of the outcome?		NA / Y / PY / PN / N / NI<br><br>NA / Y / PY / PN / N / NI
	2.4. Do start of follow-up and start of intervention coincide for most participants?		Y / PY / PN / N / NI
	2.5. **If Y/PY to 2.2 and 2.3, or N/PN to 2.4**: Were adjustment techniques used that are likely to correct for the presence of selection biases?		NA / Y / PY / PN / N / NI
	Risk of bias judgement		**Low** / Moderate / Serious / Critical / NI
	Optional: What is the predicted direction of bias due to selection of participants into the study?		Favours experimental / Favours comparator / Towards null /Away from null / Unpredictable
	**3. Bias in classification of interventions**		
	3.1 Were intervention groups clearly defined?		Y / PY / PN / N / NI

	3.2 Was the information used to define intervention groups recorded at the start of the intervention?		Y / PY / PN / N / NI
	3.3 Could classification of intervention status have been affected by knowledge of the outcome or risk of the outcome?		Y / PY / PN / N / NI
	Risk of bias judgement		**Low** / Moderate / Serious / Critical / NI
	Optional: What is the predicted direction of bias due to classification of interventions?		**Favours experimental** / Favours comparator / Towards null /Away from null / Unpredictable
	**4. Bias due to deviations from intended interventions**		
	If your aim for this study is to assess the effect of assignment to intervention, answer questions 4.1 and 4.2		
	4.1. Were there deviations from the intended intervention beyond what would be expected in usual practice?	All interventions were performed according to clinical practice.	Y / PY / PN / N / NI
	4.2. **If Y/PY to 4.1**: Were these deviations from intended intervention unbalanced between groups *and* likely to have affected the outcome?		NA / Y / PY / PN / N / NI
	If your aim for this study is to assess the effect of starting and adhering to intervention, answer questions 4.3 to 4.6		

	4.3. Were important co-interventions balanced across intervention groups?		Y / PY / PN / N / NI
	4.4. Was the intervention implemented successfully for most participants?		Y / PY / PN / N / NI
	4.5. Did study participants adhere to the assigned intervention regimen?		Y / PY / PN / N / NI
	4.6. **If N/PN to 4.3, 4.4 or 4.5**: Was an appropriate analysis used to estimate the effect of starting and adhering to the intervention?		NA / Y / PY / PN / N / NI
	Risk of bias judgement		**Low** / Moderate / Serious / Critical / NI
	Optional: What is the predicted direction of bias due to deviations from the intended interventions?		**Favours experimental** / Favours comparator / Towards null /Away from null / Unpredictable
	**5. Bias due to missing data**		
	5.1 Were outcome data available for all, or nearly all, participants?		Y / PY / PN / N / NI

	5.2 Were participants excluded due to missing data on intervention status?	Two patients Missed follow up appointment	Y / PY / PN / N / NI
	5.3 Were participants excluded due to missing data on other variables needed for the analysis?		Y / PY / PN / N / NI
	5.4 **If PN/N to 5.1, or Y/PY to 5.2 or 5.3**: Are the proportion of participants and reasons for missing data similar across interventions?		NA / Y / PY / PN / N / NI
	5.5 **If PN/N to 5.1, or Y/PY to 5.2 or 5.3**: Is there evidence that results were robust to the presence of missing data?		NA / Y / PY / PN / N / NI
	Risk of bias judgement		**Low** / Moderate / Serious / Critical / NI
	Optional: What is the predicted direction of bias due to missing data?		**Favours experimental** / Favours comparator / Towards null /Away from null / Unpredictable
	**6. Bias in measurement of outcomes**		
	6.1 Could the outcome measure have been influenced by knowledge of the intervention received?		Y / PY / PN / N / NI

	6.2 Were outcome assessors aware of the intervention received by study participants?	prospective study.	Y / PY / PN / N / NI
	6.3 Were the methods of outcome assessment comparable across intervention groups?		Y / PY / PN / N / NI
	6.4 Were any systematic errors in measurement of the outcome related to intervention received?		Y / PY / PN / N / NI
	Risk of bias judgement		**<u>Low</u>** / Moderate / Serious / Critical / NI
	Optional: What is the predicted direction of bias due to measurement of outcomes?		**<u>Favours experimental</u>** / Favours comparator / Towards null /Away from null / Unpredictable
	**7. Bias in selection of the reported result**		
	Is the reported effect estimate likely to be selected, on the basis of the results, from...		
	7.1. ... multiple outcome *measurements* within the outcome domain?		Y / PY / PN / <u>N</u> / NI

	7.2 ... multiple *analyses* of the intervention-outcome relationship?		Y / PY / PN / N / NI
	7.3 ... different *subgroups*?		Y / PY / PN / N / NI
	Risk of bias judgement		**Low** / Moderate / Serious / Critical / NI
	Optional: What is the predicted direction of bias due to selection of the reported result?		**Favours experimental** / Favours comparator / Towards null /Away from null / Unpredictable
	**Overall bias**		
	Risk of bias judgement		**Low** / Moderate / Serious / Critical / NI

	Optional: What is the overall predicted direction of bias for this outcome?		**Favours experimental** / Favours comparator / Towards null /Away from null / Unpredictable
Gurdan et al., 2018 [14]	**1. Bias due to confounding**		
	1.1 Is there potential for confounding of the effect of intervention in this study?<br><br>**If N/PN to 1.1:** the study can be considered to be at low risk of bias due to confounding and no further signalling questions need be considered		Y / PY / PN / N
	**If Y/PY to 1.1**: determine whether there is a need to assess time-varying confounding:		
	1.2. Was the analysis based on splitting participants' follow up time according to intervention received?<br><br>**If N/PN**, answer questions relating to baseline confounding (1.4 to 1.6)<br><br>**If Y/PY**, go to question 1.3.<br><br>1.3. Were intervention discontinuations or switches likely to be related to factors that are prognostic for the outcome?<br><br>**If N/PN**, answer questions relating to baseline confounding (1.4 to 1.6)<br><br>**If Y/PY**, answer questions relating to both baseline and time-varying confounding (1.7 and 1.8)		NA / Y / PY / PN / N / NI<br><br>NA / Y / PY / PN / N / NI
	Questions relating to baseline confounding only		

	1.4. Did the authors use an appropriate analysis method that controlled for all the important confounding domains?		NA / Y / PY / PN / N / NI
	1.5. **If** Y/PY **to 1.4**: Were confounding domains that were controlled for measured validly and reliably by the variables available in this study?		NA / Y / PY / PN / N / NI
	1.6. Did the authors control for any post-intervention variables that could have been affected by the intervention?		NA / Y / PY / PN / N / NI
	Questions relating to baseline and time-varying confounding		
	1.7. Did the authors use an appropriate analysis method that controlled for all the important confounding domains and for time-varying confounding?		NA / Y / PY / PN / N / NI
	1.8. **If** Y/PY **to 1.7**: Were confounding domains that were controlled for measured validly and reliably by the variables available in this study?		NA / Y / PY / PN / N / NI
	Risk of bias judgement		**Low** / Moderate / Serious / Critical / NI
	Optional: What is the predicted direction of bias due to confounding?		**Favours experimental** / Favours comparator / Unpredictable
	**2. Bias in selection of participants into the study**		

	2.1. Was selection of participants into the study (or into the analysis) based on participant characteristics observed after the start of intervention?<br><br>**If N/PN to 2.1:** go to 2.4	Selection of participants took place after start of intervention, as it was a retrospective study.	Y / PY / PN / N / NI
	2.2. **If Y/PY to 2.1**: Were the post-intervention variables that influenced selection likely to be associated with intervention?<br><br>2.3 **If Y/PY to 2.2**: Were the post-intervention variables that influenced selection likely to be influenced by the outcome or a cause of the outcome?		NA / Y / PY / PN / N / NI<br><br>NA / Y / PY / PN / N / NI
	2.4. Do start of follow-up and start of intervention coincide for most participants?		Y/ PY / PN / N / NI
	2.5. **If Y/PY to 2.2 and 2.3, or N/PN to 2.4**: Were adjustment techniques used that are likely to correct for the presence of selection biases?		NA / Y / PY / PN / N / NI
	Risk of bias judgement		**Low** / Moderate / Serious / Critical / NI
	Optional: What is the predicted direction of bias due to selection of participants into the study?		**Favours experimental** / Favours comparator / Towards null /Away from null / Unpredictable

	**3. Bias in classification of interventions**		
	3.1 Were intervention groups clearly defined?		Y / PY / PN / N / NI
	3.2 Was the information used to define intervention groups recorded at the start of the intervention?		Y / PY / PN / N / NI
	3.3 Could classification of intervention status have been affected by knowledge of the outcome or risk of the outcome?		Y / PY / PN / N / NI
	Risk of bias judgement		**Low** / Moderate / Serious / Critical / NI
	Optional: What is the predicted direction of bias due to classification of interventions?		**Favours experimental** / Favours comparator / Towards null /Away from null / Unpredictable
	**4. Bias due to deviations from intended interventions**		
	If your aim for this study is to assess the effect of assignment to intervention, answer questions 4.1 and 4.2		
	4.1. Were there deviations from the intended intervention beyond what would be expected in usual practice?	All interventions were performed according to clinical practice.	Y / PY / PN / N / NI

	4.2. **If Y/PY to 4.1**: Were these deviations from intended intervention unbalanced between groups *and* likely to have affected the outcome?		NA / Y / PY / PN / N / NI
	If your aim for this study is to assess the effect of starting and adhering to intervention, answer questions 4.3 to 4.6		
	4.3. Were important co-interventions balanced across intervention groups?		Y / PY / PN / N / NI
	4.4. Was the intervention implemented successfully for most participants?		Y / PY / PN / N / NI
	4.5. Did study participants adhere to the assigned intervention regimen?		Y / PY / PN / N / NI
	4.6. **If N/PN to 4.3, 4.4 or 4.5**: Was an appropriate analysis used to estimate the effect of starting and adhering to the intervention?		NA / Y / PY / PN / N / NI
	Risk of bias judgement		**Low** / Moderate / Serious / Critical / NI
	Optional: What is the predicted direction of bias due to deviations from the intended interventions?		**Favours experimental** / Favours comparator / Towards null /Away from null / Unpredictable
	**5. Bias due to missing data**		

	5.1 Were outcome data available for all, or nearly all, participants?		Y / PY / PN / N / NI
	5.2 Were participants excluded due to missing data on intervention status?		Y / PY / PN / N / NI
	5.3 Were participants excluded due to missing data on other variables needed for the analysis?		Y / PY / PN / N / NI
	5.4 **If PN/N to 5.1, or Y/PY to 5.2 or 5.3**: Are the proportion of participants and reasons for missing data similar across interventions?		NA / Y / PY / PN / N / NI
	5.5 **If PN/N to 5.1, or Y/PY to 5.2 or 5.3**: Is there evidence that results were robust to the presence of missing data?		NA / Y / PY / PN / N / NI
	Risk of bias judgement		**Low** / Moderate / Serious / Critical / NI
	Optional: What is the predicted direction of bias due to missing data?		**Favours experimental** / Favours comparator / Towards null /Away from null / Unpredictable
	**6. Bias in measurement of outcomes**		

	6.1 Could the outcome measure have been influenced by knowledge of the intervention received?		Y / PY / PN / N / NI
	6.2 Were outcome assessors aware of the intervention received by study participants?	Retrospective study.	Y / PY / PN / N / NI
	6.3 Were the methods of outcome assessment comparable across intervention groups?		Y / PY / PN / N / NI
	6.4 Were any systematic errors in measurement of the outcome related to intervention received?		Y / PY / PN / N / NI
	Risk of bias judgement		**Low** / Moderate / Serious / Critical / NI
	Optional: What is the predicted direction of bias due to measurement of outcomes?		**Favours experimental** / Favours comparator / Towards null /Away from null / Unpredictable
	**7. Bias in selection of the reported result**		
	Is the reported effect estimate likely to be selected, on the basis of the results, from...		

	7.1. ... multiple outcome *measurements* within the outcome domain?		Y / PY / PN / <u>N</u> / NI
	7.2 ... multiple *analyses* of the intervention-outcome relationship?		Y / PY / PN / <u>N</u> / NI
	7.3 ... different *subgroups*?		Y / PY / PN / <u>N</u> / NI
	Risk of bias judgement		**<u>Low</u>** / Moderate / Serious / Critical / NI
	Optional: What is the predicted direction of bias due to selection of the reported result?		**<u>Favours experimental</u>** / Favours comparator / Towards null /Away from null / Unpredictable
	**Overall bias**		
	Risk of bias judgement		**<u>Low</u>** / Moderate / Serious / Critical / NI

	Optional: What is the overall predicted direction of bias for this outcome?		**Favours experimental** / Favours comparator / Towards null /Away from null / Unpredictable
Hourfar et al., 2017 [15]	**1. Bias due to confounding**		
	1.1 Is there potential for confounding of the effect of intervention in this study?<br><br>**If N/PN to 1.1:** the study can be considered to be at low risk of bias due to confounding and no further signalling questions need be considered		Y / PY / PN / N
	**If Y/PY to 1.1**: determine whether there is a need to assess time-varying confounding:		
	1.2. Was the analysis based on splitting participants' follow up time according to intervention received?<br><br>**If N/PN**, answer questions relating to baseline confounding (1.4 to 1.6)<br><br>**If Y/PY**, go to question 1.3.<br><br>1.3. Were intervention discontinuations or switches likely to be related to factors that are prognostic for the outcome?<br><br>**If N/PN**, answer questions relating to baseline confounding (1.4 to 1.6)<br><br>**If Y/PY**, answer questions relating to both baseline and time-varying confounding (1.7 and 1.8)		NA / Y / PY / PN / N / NI<br><br>NA / Y / PY / PN / N / NI
	Questions relating to baseline confounding only		

	1.4. Did the authors use an appropriate analysis method that controlled for all the important confounding domains?		NA / Y / PY / PN / N / NI
	1.5. **If** Y/PY **to 1.4**: Were confounding domains that were controlled for measured validly and reliably by the variables available in this study?		NA / Y / PY / PN / N / NI
	1.6. Did the authors control for any post-intervention variables that could have been affected by the intervention?		NA / Y / PY / PN / N / NI
	Questions relating to baseline and time-varying confounding		
	1.7. Did the authors use an appropriate analysis method that controlled for all the important confounding domains and for time-varying confounding?		NA / Y / PY / PN / N / NI
	1.8. **If** Y/PY **to 1.7**: Were confounding domains that were controlled for measured validly and reliably by the variables available in this study?		NA / Y / PY / PN / N / NI
	Risk of bias judgement		**Low** / Moderate / Serious / Critical / NI
	Optional: What is the predicted direction of bias due to confounding?		**Favours experimental** / Favours comparator / Unpredictable
	**2. Bias in selection of participants into the study**		

	2.1. Was selection of participants into the study (or into the analysis) based on participant characteristics observed after the start of intervention?<br><br>**If** N/PN **to 2.1:** go to 2.4	Selection of participants took place after start of intervention, as it was a retrospective study.	Y / PY / PN / N / NI
	2.2. **If** Y/PY **to 2.1**: Were the post-intervention variables that influenced selection likely to be associated with intervention?<br><br>2.3 **If** Y/PY **to 2.2**: Were the post-intervention variables that influenced selection likely to be influenced by the outcome or a cause of the outcome?		NA / Y / PY / PN / N / NI<br><br>NA / Y / PY / PN / N / NI
	2.4. Do start of follow-up and start of intervention coincide for most participants?		Y/ PY / PN / N / NI
	2.5. **If** Y/PY **to 2.2 and 2.3, or** N/PN **to 2.4**: Were adjustment techniques used that are likely to correct for the presence of selection biases?		NA / Y / PY / PN / N / NI
	Risk of bias judgement		**Low** / Moderate / Serious / Critical / NI
	Optional: What is the predicted direction of bias due to selection of participants into the study?		**Favours experimental** / Favours comparator / Towards null /Away from null / Unpredictable

	**3. Bias in classification of interventions**		
	3.1 Were intervention groups clearly defined?		Y / PY / PN / N / NI
	3.2 Was the information used to define intervention groups recorded at the start of the intervention?		Y / PY / PN / N / NI
	3.3 Could classification of intervention status have been affected by knowledge of the outcome or risk of the outcome?		Y / PY / PN / N / NI
	Risk of bias judgement		**Low** / Moderate / Serious / Critical / NI
	Optional: What is the predicted direction of bias due to classification of interventions?		**Favours experimental** / Favours comparator / Towards null /Away from null / Unpredictable
	**4. Bias due to deviations from intended interventions**		
	If your aim for this study is to assess the effect of assignment to intervention, answer questions 4.1 and 4.2		
	4.1. Were there deviations from the intended intervention beyond what would be expected in usual practice?	All interventions were performed according to clinical practice.	Y / PY / PN / N / NI

	4.2. **If Y/PY to 4.1**: Were these deviations from intended intervention unbalanced between groups *and* likely to have affected the outcome?		NA / Y / PY / PN / N / NI
	If your aim for this study is to assess the effect of starting and adhering to intervention, answer questions 4.3 to 4.6		
	4.3. Were important co-interventions balanced across intervention groups?		Y / PY / PN / N / NI
	4.4. Was the intervention implemented successfully for most participants?		Y / PY / PN / N / NI
	4.5. Did study participants adhere to the assigned intervention regimen?		Y / PY / PN / N / NI
	4.6. **If N/PN to 4.3, 4.4 or 4.5**: Was an appropriate analysis used to estimate the effect of starting and adhering to the intervention?		NA / Y / PY / PN / N / NI
	Risk of bias judgement		**Low** / Moderate / Serious / Critical / NI
	Optional: What is the predicted direction of bias due to deviations from the intended interventions?		**Favours experimental** / Favours comparator / Towards null /Away from null / Unpredictable
	**5. Bias due to missing data**		

	5.1 Were outcome data available for all, or nearly all, participants?		Y / PY / PN / N / NI
	5.2 Were participants excluded due to missing data on intervention status?		Y / PY / PN / N / NI
	5.3 Were participants excluded due to missing data on other variables needed for the analysis?		Y / PY / PN / N / NI
	5.4 **If PN/N to 5.1, or Y/PY to 5.2 or 5.3**: Are the proportion of participants and reasons for missing data similar across interventions?		NA / Y / PY / PN / N / NI
	5.5 **If PN/N to 5.1, or Y/PY to 5.2 or 5.3**: Is there evidence that results were robust to the presence of missing data?		NA / Y / PY / PN / N / NI
	Risk of bias judgement		**Low** / Moderate / Serious / Critical / NI
	Optional: What is the predicted direction of bias due to missing data?		**Favours experimental** / Favours comparator / Towards null /Away from null / Unpredictable
	**6. Bias in measurement of outcomes**		

	6.1 Could the outcome measure have been influenced by knowledge of the intervention received?		Y / PY / PN / N / NI
	6.2 Were outcome assessors aware of the intervention received by study participants?	Retrospective study.	Y / PY / PN / N / NI
	6.3 Were the methods of outcome assessment comparable across intervention groups?		Y / PY / PN / N / NI
	6.4 Were any systematic errors in measurement of the outcome related to intervention received?		Y / PY / PN / N / NI
	Risk of bias judgement		**Low** / Moderate / Serious / Critical / NI
	Optional: What is the predicted direction of bias due to measurement of outcomes?		**Favours experimental** / Favours comparator / Towards null /Away from null / Unpredictable
	**7. Bias in selection of the reported result**		
	Is the reported effect estimate likely to be selected, on the basis of the results, from...		

	7.1. ... multiple outcome *measurements* within the outcome domain?		Y / PY / PN / N / NI
	7.2 ... multiple *analyses* of the intervention-outcome relationship?		Y / PY / PN / N / NI
	7.3 ... different *subgroups*?		Y / PY / PN / N / NI
	Risk of bias judgement		**Low** / Moderate / Serious / Critical / NI
	Optional: What is the predicted direction of bias due to selection of the reported result?		**Favours experimental** / Favours comparator / Towards null /Away from null / Unpredictable
	**Overall bias**		
	Risk of bias judgement		**Low** / Moderate / Serious / Critical / NI

	Optional: What is the overall predicted direction of bias for this outcome?		**Favours experimental** / Favours comparator / Towards null /Away from null / Unpredictable
Jung et al., 2015 [16]	**1. Bias due to confounding**		
	1.1 Is there potential for confounding of the effect of intervention in this study?<br><br>**If N/PN to 1.1:** the study can be considered to be at low risk of bias due to confounding and no further signalling questions need be considered	Regular maintenance care was provided to prevent biological, mechanical, and technical complications.	Y / PY / PN / N
	**If Y/PY to 1.1**: determine whether there is a need to assess time-varying confounding:		
	1.2. Was the analysis based on splitting participants' follow up time according to intervention received?<br><br>**If N/PN**, answer questions relating to baseline confounding (1.4 to 1.6)<br><br>**If Y/PY**, go to question 1.3.		NA / Y / PY / PN / N / NI
	1.3. Were intervention discontinuations or switches likely to be related to factors that are prognostic for the outcome?<br><br>**If N/PN**, answer questions relating to baseline confounding (1.4 to 1.6)<br><br>**If Y/PY**, answer questions relating to both baseline and time-varying confounding (1.7 and 1.8)		NA / Y / PY / PN / N / NI

	Questions relating to baseline confounding only		
	1.4. Did the authors use an appropriate analysis method that controlled for all the important confounding domains?		NA / Y / PY / PN / N / NI
	1.5. **If** <u>Y/PY</u> **to 1.4**: Were confounding domains that were controlled for measured validly and reliably by the variables available in this study?		NA / Y / PY / PN / N / NI
	1.6. Did the authors control for any post-intervention variables that could have been affected by the intervention?		NA / Y / PY / PN / N / NI
	Questions relating to baseline and time-varying confounding		
	1.7. Did the authors use an appropriate analysis method that controlled for all the important confounding domains and for time-varying confounding?		NA / Y / PY / PN / N / NI
	1.8. **If** <u>Y/PY</u> **to 1.7**: Were confounding domains that were controlled for measured validly and reliably by the variables available in this study?		NA / Y / PY / PN / N / NI
	Risk of bias judgement		<u>**Low**</u> / Moderate / Serious / Critical / NI
	Optional: What is the predicted direction of bias due to confounding?		<u>**Favours experimental**</u> / Favours comparator / Unpredictable
	**2. Bias in selection of participants into the study**		

	2.1. Was selection of participants into the study (or into the analysis) based on participant characteristics observed after the start of intervention?<br><br>**If N/PN to 2.1:** go to 2.4	Selection of participants took place after start of intervention, as it was a retrospective study.	Y / PY / PN / N / NI
	2.2. **If Y/PY to 2.1**: Were the post-intervention variables that influenced selection likely to be associated with intervention?<br><br>2.3 **If Y/PY to 2.2**: Were the post-intervention variables that influenced selection likely to be influenced by the outcome or a cause of the outcome?		NA / Y / PY / PN / N / NI<br><br>NA / Y / PY / PN / N / NI
	2.4. Do start of follow-up and start of intervention coincide for most participants?		Y / PY / PN / N / NI
	2.5. **If Y/PY to 2.2 and 2.3, or N/PN to 2.4**: Were adjustment techniques used that are likely to correct for the presence of selection biases?		NA / Y / PY / PN / N / NI
	Risk of bias judgement		**Low** / Moderate / Serious / Critical / NI
	Optional: What is the predicted direction of bias due to selection of participants into the study?		**Favours experimental** / Favours comparator / Towards null /Away from null / Unpredictable

	**3. Bias in classification of interventions**		
	3.1 Were intervention groups clearly defined?	Fractured dental implants.	Y / PY / PN / N / NI
	3.2 Was the information used to define intervention groups recorded at the start of the intervention?		Y / PY / PN / N / NI
	3.3 Could classification of intervention status have been affected by knowledge of the outcome or risk of the outcome?	The analysis was limited to assessing the pattern of implant fractures and clinical factors.	Y / PY / PN / N / NI
	Risk of bias judgement		**Low** / Moderate / Serious / Critical / NI
	Optional: What is the predicted direction of bias due to classification of interventions?		**Favours experimental** / Favours comparator / Towards null /Away from null / Unpredictable
	**4. Bias due to deviations from intended interventions**		
	If your aim for this study is to assess the effect of assignment to intervention, answer questions 4.1 and 4.2		

	4.1. Were there deviations from the intended intervention beyond what would be expected in usual practice?	All interventions were performed according to clinical practice.	Y / PY / PN / N / NI
	4.2. **If Y/PY to 4.1**: Were these deviations from intended intervention unbalanced between groups *and* likely to have affected the outcome?		NA / Y / PY / PN / N / NI
	If your aim for this study is to assess the effect of starting and adhering to intervention, answer questions 4.3 to 4.6		
	4.3. Were important co-interventions balanced across intervention groups?		Y / PY / PN / N / NI
	4.4. Was the intervention implemented successfully for most participants?		Y / PY / PN / N / NI
	4.5. Did study participants adhere to the assigned intervention regimen?		Y / PY / PN / N / NI
	4.6. **If N/PN to 4.3, 4.4 or 4.5**: Was an appropriate analysis used to estimate the effect of starting and adhering to the intervention?		NA / Y / PY / PN / N / NI
	Risk of bias judgement		**Low** / Moderate / Serious / Critical / NI

	Optional: What is the predicted direction of bias due to deviations from the intended interventions?		**Favours experimental** / Favours comparator / Towards null /Away from null / Unpredictable
	**5. Bias due to missing data**		
	5.1 Were outcome data available for all, or nearly all, participants?		Y / PY / PN / N / NI
	5.2 Were participants excluded due to missing data on intervention status?		Y / PY / PN / N / NI
	5.3 Were participants excluded due to missing data on other variables needed for the analysis?		Y / PY / PN / N / NI
	5.4 **If PN/N to 5.1, or Y/PY to 5.2 or 5.3**: Are the proportion of participants and reasons for missing data similar across interventions?		NA / Y / PY / PN / N / NI
	5.5 **If PN/N to 5.1, or Y/PY to 5.2 or 5.3**: Is there evidence that results were robust to the presence of missing data?		NA / Y / PY / PN / N / NI
	Risk of bias judgement		**Low** / Moderate / Serious / Critical / NI

	Optional: What is the predicted direction of bias due to missing data?		**Favours experimental** / Favours comparator / Towards null /Away from null / Unpredictable
	**6. Bias in measurement of outcomes**		
	6.1 Could the outcome measure have been influenced by knowledge of the intervention received?		Y / PY / PN / N / NI
	6.2 Were outcome assessors aware of the intervention received by study participants?	Retrospective study.	Y / PY / PN / N / NI
	6.3 Were the methods of outcome assessment comparable across intervention groups?		Y / PY / PN / N / NI
	6.4 Were any systematic errors in measurement of the outcome related to intervention received?		Y / PY / PN / N / NI
	Risk of bias judgement		**Low** / Moderate / Serious / Critical / NI

	Optional: What is the predicted direction of bias due to measurement of outcomes?		**Favours experimental** / Favours comparator / Towards null /Away from null / Unpredictable
	**7. Bias in selection of the reported result**		
	Is the reported effect estimate likely to be selected, on the basis of the results, from...		
	7.1. ... multiple outcome *measurements* within the outcome domain?		Y / PY / PN / N / NI
	7.2 ... multiple *analyses* of the intervention-outcome relationship?		Y / PY / PN / N / NI
	7.3 ... different *subgroups*?		Y / PY / PN / N / NI
	Risk of bias judgement		**Low** / Moderate / Serious / Critical / NI

	Optional: What is the predicted direction of bias due to selection of the reported result?		**Favours experimental** / Favours comparator / Towards null /Away from null / Unpredictable
	**Overall bias**		
	Risk of bias judgement		**Low** / Moderate / Serious / Critical / NI
	Optional: What is the overall predicted direction of bias for this outcome?		**Favours experimental** / Favours comparator / Towards null /Away from null / Unpredictable
Jia et al., 2018 [17]	**1. Bias due to confounding**		
	1.1 Is there potential for confounding of the effect of intervention in this study?<br><br>**If N/PN to 1.1:** the study can be considered to be at low risk of bias due to confounding and no further signalling questions need be considered	All patients who experienced mechanical complications were examined for presence of parafunctional activity.	Y / PY / PN / N

	**If Y/PY to 1.1**: determine whether there is a need to assess time-varying confounding:		
	1.2. Was the analysis based on splitting participants' follow up time according to intervention received?<br><br>**If N/PN**, answer questions relating to baseline confounding (1.4 to 1.6)<br><br>**If Y/PY**, go to question 1.3.<br><br>1.3. Were intervention discontinuations or switches likely to be related to factors that are prognostic for the outcome?<br><br>**If N/PN**, answer questions relating to baseline confounding (1.4 to 1.6)<br><br>**If Y/PY**, answer questions relating to both baseline and time-varying confounding (1.7 and 1.8)		NA / Y / PY / PN / N / NI<br><br><br><br><br><br>NA / Y / PY / PN / N / NI
	Questions relating to baseline confounding only		
	1.4. Did the authors use an appropriate analysis method that controlled for all the important confounding domains?		NA / Y / PY / PN / N / NI
	1.5. **If Y/PY to 1.4**: Were confounding domains that were controlled for measured validly and reliably by the variables available in this study?		NA / Y / PY / PN / N / NI
	1.6. Did the authors control for any post-intervention variables that could have been affected by the intervention?		NA / Y / PY / PN / N / NI
	Questions relating to baseline and time-varying confounding		
	1.7. Did the authors use an appropriate analysis method that controlled for all the important confounding domains and for time-varying confounding?		NA / Y / PY / PN / N / NI

	1.8. **If Y/PY to 1.7**: Were confounding domains that were controlled for measured validly and reliably by the variables available in this study?		NA / Y / PY / PN / N / NI
	Risk of bias judgement		**Low** / Moderate / Serious / Critical / NI
	Optional: What is the predicted direction of bias due to confounding?		**Favours experimental** / Favours comparator / Unpredictable
	**2. Bias in selection of participants into the study**		
	2.1. Was selection of participants into the study (or into the analysis) based on participant characteristics observed after the start of intervention?<br><br>**If N/PN to 2.1:** go to 2.4	Selection of participants took place after start of intervention, as it was a retrospective study.	Y / PY / PN / N / NI
	2.2. **If Y/PY to 2.1**: Were the post-intervention variables that influenced selection likely to be associated with intervention?<br><br>2.3 **If Y/PY to 2.2**: Were the post-intervention variables that influenced selection likely to be influenced by the outcome or a cause of the outcome?		NA / Y / PY / PN / N / NI<br><br>NA / Y / PY / PN / N / NI
	2.4. Do start of follow-up and start of intervention coincide for most participants?		Y / PY / PN / N / NI

	2.5. **If Y/PY to 2.2 and 2.3, or N/PN to 2.4**: Were adjustment techniques used that are likely to correct for the presence of selection biases?		NA / Y / PY / PN / N / NI
	Risk of bias judgement		**Low** / Moderate / Serious / Critical / NI
	Optional: What is the predicted direction of bias due to selection of participants into the study?		**Favours experimental** / Favours comparator / Towards null /Away from null / Unpredictable
	**3. Bias in classification of interventions**		
	3.1 Were intervention groups clearly defined?		Y / PY / PN / N / NI
	3.2 Was the information used to define intervention groups recorded at the start of the intervention?		Y / PY / PN / N / NI
	3.3 Could classification of intervention status have been affected by knowledge of the outcome or risk of the outcome?		Y / PY / PN / N / NI
	Risk of bias judgement		**Low** / Moderate / Serious / Critical / NI

	Optional: What is the predicted direction of bias due to classification of interventions?		**Favours experimental** / Favours comparator / Towards null /Away from null / Unpredictable
	**4. Bias due to deviations from intended interventions**		
	If your aim for this study is to assess the effect of assignment to intervention, answer questions 4.1 and 4.2		
	4.1. Were there deviations from the intended intervention beyond what would be expected in usual practice?	All interventions were performed according to clinical practice.	Y / PY / PN / N / NI
	4.2. **If Y/PY to 4.1**: Were these deviations from intended intervention unbalanced between groups *and* likely to have affected the outcome?		NA / Y / PY / PN / N / NI
	If your aim for this study is to assess the effect of starting and adhering to intervention, answer questions 4.3 to 4.6		
	4.3. Were important co-interventions balanced across intervention groups?		Y / PY / PN / N / NI
	4.4. Was the intervention implemented successfully for most participants?		Y / PY / PN / N / NI
	4.5. Did study participants adhere to the assigned intervention regimen?		Y / PY / PN / N / NI
	4.6. **If N/PN to 4.3, 4.4 or 4.5**: Was an appropriate analysis used to estimate the effect of starting and adhering to the intervention?		NA / Y / PY / PN / N / NI

	Risk of bias judgement		**Low** / Moderate / Serious / Critical / NI
	Optional: What is the predicted direction of bias due to deviations from the intended interventions?		**Favours experimental** / Favours comparator / Towards null /Away from null / Unpredictable
	**5. Bias due to missing data**		
	5.1 Were outcome data available for all, or nearly all, participants?		Y / PY / PN / N / NI
	5.2 Were participants excluded due to missing data on intervention status?		Y / PY / PN / N / NI
	5.3 Were participants excluded due to missing data on other variables needed for the analysis?		Y / PY / PN / N / NI
	5.4 **If PN/N to 5.1, or Y/PY to 5.2 or 5.3**: Are the proportion of participants and reasons for missing data similar across interventions?		NA / Y / PY / PN / N / NI
	5.5 **If PN/N to 5.1, or Y/PY to 5.2 or 5.3**: Is there evidence that results were robust to the presence of missing data?		NA / Y / PY / PN / N / NI

	Risk of bias judgement		**Low** / Moderate / Serious / Critical / NI
	Optional: What is the predicted direction of bias due to missing data?		**Favours experimental** / Favours comparator / Towards null /Away from null / Unpredictable
	**6. Bias in measurement of outcomes**		
	6.1 Could the outcome measure have been influenced by knowledge of the intervention received?		Y / PY / PN / N / NI
	6.2 Were outcome assessors aware of the intervention received by study participants?	Retrospective study.	Y / PY / PN / N / NI
	6.3 Were the methods of outcome assessment comparable across intervention groups?		Y / PY / PN / N / NI
	6.4 Were any systematic errors in measurement of the outcome related to intervention received?		Y / PY / PN / N / NI
	Risk of bias judgement		**Low** / Moderate / Serious / Critical / NI

	Optional: What is the predicted direction of bias due to measurement of outcomes?		**<u>Favours experimental</u>** / Favours comparator / Towards null /Away from null / Unpredictable
	**7. Bias in selection of the reported result**		
	Is the reported effect estimate likely to be selected, on the basis of the results, from...		
	7.1. ... multiple outcome *measurements* within the outcome domain?		Y / PY / PN / N / NI
	7.2 ... multiple *analyses* of the intervention-outcome relationship?		Y / PY / PN / N / NI
	7.3 ... different *subgroups*?		Y / PY / PN / N / NI
	Risk of bias judgement		**<u>Low</u>** / Moderate / Serious / Critical / NI

	Optional: What is the predicted direction of bias due to selection of the reported result?		**Favours experimental** / Favours comparator / Towards null /Away from null / Unpredictable
	**Overall bias**		
	Risk of bias judgement		**Low** / Moderate / Serious / Critical / NI
	Optional: What is the overall predicted direction of bias for this outcome?		**Favours experimental** / Favours comparator / Towards null /Away from null / Unpredictable
Takaki et al., 2010 [18]	**1. Bias due to confounding**		
	1.1 Is there potential for confounding of the effect of intervention in this study?<br>**If N/PN to 1.1:** the study can be considered to be at low risk of bias due to confounding and no further signalling questions need be considered		Y / PY / PN / N

	**If Y/PY to 1.1**: determine whether there is a need to assess time-varying confounding:		
	1.2. Was the analysis based on splitting participants' follow up time according to intervention received?<br><br>**If N/PN**, answer questions relating to baseline confounding (1.4 to 1.6)<br><br>**If Y/PY**, go to question 1.3.<br><br>1.3. Were intervention discontinuations or switches likely to be related to factors that are prognostic for the outcome?<br><br>**If N/PN**, answer questions relating to baseline confounding (1.4 to 1.6)<br><br>**If Y/PY**, answer questions relating to both baseline and time-varying confounding (1.7 and 1.8)		NA / Y / PY / PN / N / NI<br><br><br><br><br><br>NA / Y / PY / PN / N / NI
	Questions relating to baseline confounding only		
	1.4. Did the authors use an appropriate analysis method that controlled for all the important confounding domains?		NA / Y / PY / PN / N / NI
	1.5. **If Y/PY to 1.4**: Were confounding domains that were controlled for measured validly and reliably by the variables available in this study?		NA / Y / PY / PN / N / NI
	1.6. Did the authors control for any post-intervention variables that could have been affected by the intervention?		NA / Y / PY / PN / N / NI
	Questions relating to baseline and time-varying confounding		
	1.7. Did the authors use an appropriate analysis method that controlled for all the important confounding domains and for time-varying confounding?		NA / Y / PY / PN / N / NI

	1.8. **If Y/PY to 1.7**: Were confounding domains that were controlled for measured validly and reliably by the variables available in this study?		NA / Y / PY / PN / N / NI
	Risk of bias judgement		**Low** / Moderate / Serious / Critical / NI
	Optional: What is the predicted direction of bias due to confounding?		**Favours experimental** / Favours comparator / Unpredictable
	**2. Bias in selection of participants into the study**		
	2.1. Was selection of participants into the study (or into the analysis) based on participant characteristics observed after the start of intervention?<br><br>**If N/PN to 2.1:** go to 2.4	Selection of participants took place after start of intervention, as it was a retrospective study.	Y / PY / PN / N / NI
	2.2. **If Y/PY to 2.1**: Were the post-intervention variables that influenced selection likely to be associated with intervention?<br><br>2.3 **If Y/PY to 2.2**: Were the post-intervention variables that influenced selection likely to be influenced by the outcome or a cause of the outcome?		NA / Y / PY / PN / N / NI<br><br>NA / Y / PY / PN / N / NI
	2.4. Do start of follow-up and start of intervention coincide for most participants?		Y / PY / PN / N / NI

	2.5. If Y/PY to 2.2 and 2.3, or N/PN to 2.4: Were adjustment techniques used that are likely to correct for the presence of selection biases?		NA / Y / PY / PN / N / NI
	Risk of bias judgement		**Low** / Moderate / Serious / Critical / NI
	Optional: What is the predicted direction of bias due to selection of participants into the study?		**Favours experimental** / Favours comparator / Towards null /Away from null / Unpredictable
	**3. Bias in classification of interventions**		
	3.1 Were intervention groups clearly defined?		Y / PY / PN / N / NI
	3.2 Was the information used to define intervention groups recorded at the start of the intervention?		Y / PY / PN / N / NI
	3.3 Could classification of intervention status have been affected by knowledge of the outcome or risk of the outcome?		Y / PY / PN / N / NI
	Risk of bias judgement		**Low** / Moderate / Serious / Critical / NI

	Optional: What is the predicted direction of bias due to classification of interventions?		**Favours experimental** / Favours comparator / Towards null /Away from null / Unpredictable
	**4. Bias due to deviations from intended interventions**		
	If your aim for this study is to assess the effect of assignment to intervention, answer questions 4.1 and 4.2		
	4.1. Were there deviations from the intended intervention beyond what would be expected in usual practice?	All interventions were performed according to clinical practice.	Y / PY / PN / N / NI
	4.2. **If Y/PY to 4.1**: Were these deviations from intended intervention unbalanced between groups *and* likely to have affected the outcome?		NA / Y / PY / PN / N / NI
	If your aim for this study is to assess the effect of starting and adhering to intervention, answer questions 4.3 to 4.6		
	4.3. Were important co-interventions balanced across intervention groups?		Y / PY / PN / N / NI
	4.4. Was the intervention implemented successfully for most participants?		Y / PY / PN / N / NI
	4.5. Did study participants adhere to the assigned intervention regimen?		Y / PY / PN / N / NI
	4.6. **If N/PN to 4.3, 4.4 or 4.5**: Was an appropriate analysis used to estimate the effect of starting and adhering to the intervention?		NA / Y / PY / PN / N / NI

	Risk of bias judgement		**Low** / Moderate / Serious / Critical / NI
	Optional: What is the predicted direction of bias due to deviations from the intended interventions?		Favours experimental / Favours comparator / Towards null /Away from null / Unpredictable
	**5. Bias due to missing data**		
	5.1 Were outcome data available for all, or nearly all, participants?		Y / PY / PN / N / NI
	5.2 Were participants excluded due to missing data on intervention status?		Y / PY / PN / N / NI
	5.3 Were participants excluded due to missing data on other variables needed for the analysis?		Y / PY / PN / N / NI
	5.4 **If PN/N to 5.1, or Y/PY to 5.2 or 5.3**: Are the proportion of participants and reasons for missing data similar across interventions?		NA / Y / PY / PN / N / NI
	5.5 **If PN/N to 5.1, or Y/PY to 5.2 or 5.3**: Is there evidence that results were robust to the presence of missing data?		NA / Y / PY / PN / N / NI

	Risk of bias judgement		**Low** / Moderate / Serious / Critical / NI
	Optional: What is the predicted direction of bias due to missing data?		**Favours experimental** / Favours comparator / Towards null /Away from null / Unpredictable
	**6. Bias in measurement of outcomes**		
	6.1 Could the outcome measure have been influenced by knowledge of the intervention received?		Y / PY / PN / N / NI
	6.2 Were outcome assessors aware of the intervention received by study participants?	Retrospective study.	Y / PY / PN / N / NI
	6.3 Were the methods of outcome assessment comparable across intervention groups?		Y / PY / PN / N / NI
	6.4 Were any systematic errors in measurement of the outcome related to intervention received?		Y / PY / PN / N / NI
	Risk of bias judgement		**Low** / Moderate / Serious / Critical / NI

	Optional: What is the predicted direction of bias due to measurement of outcomes?		**Favours experimental** / Favours comparator / Towards null /Away from null / Unpredictable
	**7. Bias in selection of the reported result**		
	Is the reported effect estimate likely to be selected, on the basis of the results, from...		
	7.1. ... multiple outcome *measurements* within the outcome domain?		Y / PY / PN / N / NI
	7.2 ... multiple *analyses* of the intervention-outcome relationship?		Y / PY / PN / N / NI
	7.3 ... different *subgroups*?		Y / PY / PN / N / NI
	Risk of bias judgement		**Low** / Moderate / Serious / Critical / NI

	Optional: What is the predicted direction of bias due to selection of the reported result?		**Favours experimental** / Favours comparator / Towards null /Away from null / Unpredictable
	**Overall bias**		
	Risk of bias judgement		**Low** / Moderate / Serious / Critical / NI
	Optional: What is the overall predicted direction of bias for this outcome?		**Favours experimental** / Favours comparator / Towards null /Away from null / Unpredictable
Ziebura et al., 2012 [19]	**1. Bias due to confounding**		

	1.1 Is there potential for confounding of the effect of intervention in this study?<br><br>**If N/PN to 1.1:** the study can be considered to be at low risk of bias due to confounding and no further signalling questions need be considered		Y / PY / PN / N
	**If Y/PY to 1.1**: determine whether there is a need to assess time-varying confounding:		
	1.2. Was the analysis based on splitting participants' follow up time according to intervention received?<br><br>**If N/PN**, answer questions relating to baseline confounding (1.4 to 1.6)<br><br>**If Y/PY**, go to question 1.3.		NA / Y / PY / PN / N / NI
	1.3. Were intervention discontinuations or switches likely to be related to factors that are prognostic for the outcome?<br><br>**If N/PN**, answer questions relating to baseline confounding (1.4 to 1.6)<br><br>**If Y/PY**, answer questions relating to both baseline and time-varying confounding (1.7 and 1.8)		NA / Y / PY / PN / N / NI
	Questions relating to baseline confounding only		
	1.4. Did the authors use an appropriate analysis method that controlled for all the important confounding domains?		NA / Y / PY / PN / N / NI
	1.5. **If Y/PY to 1.4**: Were confounding domains that were controlled for measured validly and reliably by the variables available in this study?		NA / Y / PY / PN / N / NI
	1.6. Did the authors control for any post-intervention variables that could have been affected by the intervention?		NA / Y / PY / PN / N / NI
	Questions relating to baseline and time-varying confounding		

	1.7. Did the authors use an appropriate analysis method that controlled for all the important confounding domains and for time-varying confounding?		NA / Y / PY / PN / N / NI
	1.8. **If Y/PY to 1.7**: Were confounding domains that were controlled for measured validly and reliably by the variables available in this study?		NA / Y / PY / PN / N / NI
	Risk of bias judgement		**Low** / Moderate / Serious / Critical / NI
	Optional: What is the predicted direction of bias due to confounding?		**Favours experimental** / Favours comparator / Unpredictable
	**2. Bias in selection of participants into the study**		
	2.1. Was selection of participants into the study (or into the analysis) based on participant characteristics observed after the start of intervention?<br><br>**If N/PN to 2.1:** go to 2.4	Selection of participants took place after start of intervention, as it was a retrospective study.	Y / PY / PN / N / NI
	2.2. **If Y/PY to 2.1**: Were the post-intervention variables that influenced selection likely to be associated with intervention?<br><br>2.3 **If Y/PY to 2.2**: Were the post-intervention variables that influenced selection likely to be influenced by the outcome or a cause of the outcome?		NA / Y / PY / PN / N / NI<br><br>NA / Y / PY / PN / N / NI

	2.4. Do start of follow-up and start of intervention coincide for most participants?		Y / PY / PN / N / NI
	2.5. **If Y/PY to 2.2 and 2.3, or N/PN to 2.4**: Were adjustment techniques used that are likely to correct for the presence of selection biases?		NA / Y / PY / PN / N / NI
	Risk of bias judgement		**Low** / Moderate / Serious / Critical / NI
	Optional: What is the predicted direction of bias due to selection of participants into the study?		**Favours experimental** / Favours comparator / Towards null /Away from null / Unpredictable
	**3. Bias in classification of interventions**		
	3.1 Were intervention groups clearly defined?	Fractured dental implants.	Y / PY / PN / N / NI
	3.2 Was the information used to define intervention groups recorded at the start of the intervention?		Y / PY / PN / N / NI
	3.3 Could classification of intervention status have been affected by knowledge of the outcome or risk of the outcome?	The analysis was limited to analyzing possible factors leading implant fractures.	Y / PY / PN / N / NI

	Risk of bias judgement		**Low** / Moderate / Serious / Critical / NI
	Optional: What is the predicted direction of bias due to classification of interventions?		**Favours experimental** / Favours comparator / Towards null /Away from null / Unpredictable
	**4. Bias due to deviations from intended interventions**		
	If your aim for this study is to assess the effect of assignment to intervention, answer questions 4.1 and 4.2		
	4.1. Were there deviations from the intended intervention beyond what would be expected in usual practice?	All interventions were performed according to clinical practice.	Y / PY / PN / N / NI
	4.2. **If Y/PY to 4.1**: Were these deviations from intended intervention unbalanced between groups *and* likely to have affected the outcome?		NA / Y / PY / PN / N / NI
	If your aim for this study is to assess the effect of starting and adhering to intervention, answer questions 4.3 to 4.6		
	4.3. Were important co-interventions balanced across intervention groups?		Y / PY / PN / N / NI

	4.4. Was the intervention implemented successfully for most participants?		Y / PY / PN / N / NI
	4.5. Did study participants adhere to the assigned intervention regimen?		Y / PY / PN / N / NI
	4.6. **If N/PN to 4.3, 4.4 or 4.5**: Was an appropriate analysis used to estimate the effect of starting and adhering to the intervention?		NA / Y / PY / PN / N / NI
	Risk of bias judgement		**Low** / Moderate / Serious / Critical / NI
	Optional: What is the predicted direction of bias due to deviations from the intended interventions?		**Favours experimental** / Favours comparator / Towards null /Away from null / Unpredictable
	**5. Bias due to missing data**		
	5.1 Were outcome data available for all, or nearly all, participants?		Y / PY / PN / N / NI
	5.2 Were participants excluded due to missing data on intervention status?		Y / PY / PN / N / NI

	5.3 Were participants excluded due to missing data on other variables needed for the analysis?		Y / PY / PN / N / NI
	5.4 **If PN/N to 5.1, or Y/PY to 5.2 or 5.3**: Are the proportion of participants and reasons for missing data similar across interventions?		NA / Y / PY / PN / N / NI
	5.5 **If PN/N to 5.1, or Y/PY to 5.2 or 5.3**: Is there evidence that results were robust to the presence of missing data?		NA / Y / PY / PN / N / NI
	Risk of bias judgement		**Low** / Moderate / Serious / Critical / NI
	Optional: What is the predicted direction of bias due to missing data?		**Favours experimental** / Favours comparator / Towards null /Away from null / Unpredictable
	**6. Bias in measurement of outcomes**		
	6.1 Could the outcome measure have been influenced by knowledge of the intervention received?		Y / PY / PN / N / NI
	6.2 Were outcome assessors aware of the intervention received by study participants?	Retrospective study.	Y / PY / PN / N / NI

	6.3 Were the methods of outcome assessment comparable across intervention groups?		Y / PY / PN / N / NI
	6.4 Were any systematic errors in measurement of the outcome related to intervention received?		Y / PY / PN / N / NI
	Risk of bias judgement		**Low** / Moderate / Serious / Critical / NI
	Optional: What is the predicted direction of bias due to measurement of outcomes?		**Favours experimental** / Favours comparator / Towards null /Away from null / Unpredictable
	**7. Bias in selection of the reported result**		
	Is the reported effect estimate likely to be selected, on the basis of the results, from...		
	7.1. ... multiple outcome *measurements* within the outcome domain?		Y / PY / PN / N / NI
	7.2 ... multiple *analyses* of the intervention-outcome relationship?		Y / PY / PN / N / NI

	7.3 … different *subgroups*?		Y / PY / PN / N / NI
	Risk of bias judgement		**Low** / Moderate / Serious / Critical / NI
	Optional: What is the predicted direction of bias due to selection of the reported result?		**Favours experimental** / Favours comparator / Towards null /Away from null / Unpredictable
	**Overall bias**		
	Risk of bias judgement		**Low** / Moderate / Serious / Critical / NI

	Optional: What is the overall predicted direction of bias for this outcome?		**Favours experimental** / Favours comparator / Towards null /Away from null / Unpredictable

Table S6: Evidence of studies included in this review.

Authors and Year of Publication	Study Design and Aim	Methods	Results	Conclusions
Golshah et al., 2021 [20]	A 6 months randomized clinical trial to assess the effect of miniscrew insertion angle (vertical and oblique) on its clinical survival under shearing forces in orthodontic patients undergoing canine retraction.	50 miniscrews were placed bilaterally in 25 patients with 45° and 90° insertion angles relative to a line perpendicular to the occlusal plane distal to the maxillary first premolar extraction site. Allocation of insertion angles to the right/left side was random using the Random Allocation Software. The patients, clinician, and statistician were blinded to the allocation of miniscrews to the side of jaw. The patients were followed-up monthly for 6 months. The primary outcome was the clinical survival of miniscrews, which was evaluated at each follow-up session. The secondary outcomes were the miniscrew stability based on the Periotest value (PTV) and the level of pain experienced by patients at 1, 12, and 24 h, and 7 days after miniscrew placement	The clinical survival rate of miniscrews placed at 90° and 45° angles was 76% and 88%, respectively. This difference was not statistically significant (P = 0.375). No significant difference was noted between the two groups regarding the PTV or the pain score either (P > 0.05).	Clinically, the insertion angle of miniscrews (90° versus 45° relative to a line perpendicular to the occlusal plane) has no significant effect on the miniscrew survival rate or stability during orthodontic treatment.

		using a visual analog scale (VAS). Data were analyzed using paired t-test, repeated measures ANOVA, and McNemar's test.		
Aboshady et al., 2022 [21]	A randomized clinical trial to to assess the failure rate after mini-screw insertion using digital three-dimensional printed guide versus free hand placement technique.	Forty-two patients with mean age (22.56 ± 3.47 years) indicated for upper first premolars' extraction (Bimaxillary protrusion and Class II division 1) were included in the study. Their maxillary quadrants were randomized to receive mini-screws as means of anchorage. Pre-operative maxillary cone-beam computed tomography scan with ultra-low-dose protocol was imaged and the maxillary arch was scanned using intra-oral scanner to obtain stereo-lithographic format file for the maxillary arch. Using *in vivo* and Rapidform Geomagic Studio® -Softwares the mini-screws were planned to be inserted in the buccal inter-radicular space between the upper second premolar and first molar in both right and left sides. For the intervention sides; digital three-dimensional guides were designed and printed for mini-screw insertion. Failure of the mini-screws was assessed till 3 months of loading.	There was no statistical significant difference in failure rate of mini-screws in both intervention (7.14%) and control sides (16.6%), with weak and moderate correlation between the root proximity and the mini-screws failure in intervention and control groups respectively.	Using a digital three-dimensional printed guide for mini-screw insertion had no effect on the failure rate of the inserted mini-screws.

Fabbroni et al., 2004 [13]	A 7-month prospective clinical study, to examine the incidence of dental damage with transalveolar screws and its sequelae.	55 patients with fractures of the mandible requiring intra- or post-operative control of their occlusion with transalveolar screws were entered in the study. Following screw removal, contact was assessed radiographically and all teeth adjacent to the screws were tested for vitality. Any contacts were judged to be minor if less than 50% of the diameter of the screw hole impinged on a tooth root or major if this was more than 50%.	There were 232 screws placed in these patients adjacent to 440 teeth. Twenty-six screws (11.2%) had major contacts and 37 (15.9%) had minor contacts. Seventeen teeth were tested as non-vital with electronic pulp testing but of these only 6 showed any impingement by screws. Two screws were associated with complications in two patients.	Screw/tooth contact does occur using transalveolar screws, however, the incidence of clinically significant damage appears to be very low.
Fäh et al., 2014 [22]	An 11 years retrospective study to assess the frequency and variety of surgical complications and adverse patient reactions associated with the implantation and explantation of palatal implants.	The implantations and explantations of palatal implants in 146 patients who had undergone orthodontic treatment using a palatal implant for anchorage in the time period 1999–2010 were evaluated retrospectively. All complications and adverse patient reactions associated with the surgical intervention of implantation and	Of the 146 palatal implants reviewed, 104 implantations and 44 explantations met the inclusion criteria and their data could be extracted. Of the 104 implantations, 25 (24.0%) surgical complications and adverse patient reactions could be documented. They consisted of lack of primary stability: 7 (6.7%),	A wide spectrum of surgical complications and adverse patient reactions after palatal implant insertion and removal was found. All complications were of minor severity and duration except after one implantation, where a prolonged hypoesthesia of the anterior palate was found. Altohugh only a small risk of permanent sensory impairment of the anterior

		explantation of the implamt were assessed.	prolonged pain: 7 (6.7%), secondary bleeding: 6 (5.8%), perforation ofnasal floor: 2 (1.9%), necrotic mucosa anterior of the implant: 2 (1.9%) and sensory impairmentof the anterior palate: 1 (1%). The respective incidents for the 44 explantations were: disturbedwound healing: 3 (6.8%), perforation of nasal floor: 1 (2.3%), secondary bleeding: 1 (2.3%) and fracture of the implant.	palatal region remains, patients must be well informed accordigly.
Gurdan et al., 2018 [14]	A 2 years retrospective study to the success and complication rates of orthodontic mini-implants.	Every patient had one or more of the 1.6 mm × 8 mm in size self-drilling mini-implants (Jeil Dual Top Anchor System, Jeil Medical Corp., Seoul, Korea). Screw loading was performed immediately after insertions, keeping tension forces under 150 g. Soft tissue and bone infections, implant mobility and screw loss, implant fracture, and neighboring tooth injury were registered. Relationships between variables were tested using the Chi-	The success rate of the orthodontic mini-implants was 89.8% in this study while the average loading period was 8.1 months. Soft-tissue infections varied between 6.3% and 33.3% of the cases while screw mobility varied between 3.1% and 20.8% of the cases regarding the anatomic localization. Screw mobility was significantly more frequent in the buccal fold than	The overall success rate of mini-implants was found acceptable in this study, however, screw mobility in the buccal fold showed a high incidence, suggesting the thorough consideration of the immediate loading by buccal mini-implants.

		square test for statistical significance.	in the palate (P = 0.034). Screw mobility was significantly more frequent in the buccal fold than in the palate (P = 0.034) and screw mobility was found more frequently in case of intrusions than by extrusions (P = 0.036).	
Hourfar et al., 2017 [15]	A 24 months retrospective study to evaluate the incidence of loss to pulp sensibility testing (PST) of maxillary front teeth after paramedian (3 to 5 mm away from the suture) orthodontic mini-implant (OMI) insertion in the anterior palate.	A total of 284 patients (102 males, 182 females; mean age was 14.4 years (±8.8) years at time of OMI-Insertion) with a total of 568 OMIs (1.7 mm diameter, length 8 mm) were retrospectively investigated. A binomial regression analysis was performed to explore covariates, such as age, gender, inclination of upper central incisors, dentition status and insertion position of OMIs that could have contributed to loss of sensibility. Statistical significance was set at $p < 0.05$.	Loss of response to PST was encountered during retention in 3 out of 284 patients and the respective OMIs had been placed at height of the second rugae (R-2). Affected teeth were a right canine, a left lateral and a left central incisor. Subsequent root canal treatment was successful. Results of the binomial regression analysis revealed that the covariate insertion position (R-2) of OMIs ($p = 0.008$) had statistically significant influence on loss of response to PST.	Although there was no radiographic evidence for direct root injury, the proximity of the implants to the anterior teeth was nevertheless statistically related to loss of PST. In all cases of PST loss OMIs were inserted at the second rugae. Therefore OMIs should be placed either more posteriorly, at the third rugae or in the median plane. Loss of PST was not increased for patients with palatal OMI (0.18%) compared to samples without OMI (0.25%).

Jung et al., 2015 [16]	A 10 months cohort study to investigate the prevalence of distinguishable soft tissue scarring after the removal of temporary anchorage devices (TADs) such as orthodontic miniscrews and to analyze the factors associated with scar formation.	The prevalence of soft tissue scarring in 66 patients (202 miniscrew removal sites) was clinically investigated at least 1 year after miniscrew removal. To determine the clinical factors associated with soft tissue scar formation, miniscrew stability; host factors including age, gender, and gingival biotype; and miniscrew-related factors such as insertion site, vertical position, and insertion period were evaluated.	The prevalence of a distinguishable scar remaining at least 1 year after miniscrew removal was 44.6%. Patients with flat gingiva showed a significantly higher prevalence of soft tissue scar formation than did those with pronounced scalloped gingiva ($P < .05$). Maxillary buccal removal sites showed a significantly higher prevalence of soft tissue scar formation than did those in the mandible or palatal slope ($P < .05$). Miniscrew sites at the alveolar mucosa showed a significantly lower prevalence of soft tissue scar formation than did those in the mucogingival junction or the attached gingiva ($P < .01$).	The prevalence of distinguishable scarring after miniscrew removal was fairly high. On the basis of our results, patients with flat gingiva and buccal interdental gingival insertion sites are more susceptible to scar formation.
Jia et al., 2018 [17]	A 35 months retrospective study to investigate the incidence of penetration of mini-implants into the sinus and the relationship between	Data from 32 patients who received mini-implants in the infrazygomatic crest were collected from a data base. The success rate of mini- implants	The overall success rate of mini-implants in the infrazygomatic crest was 96.7%, and 78.3% penetrated into the sinus. In	The incidence of penetration of infrazygomatic crest mini- implants into the sinus may be high. Penetration through double cortical bone plates with limitation of the

	penetration depth and sinus tissue.	was determined by clinical retrospective analysis. The incidence of penetration, penetration depth, and sinus configuration were investigated and compared between cone-beam computed tomography scans obtained immediately after insertion and before mini-implant removal.	the group in which penetration exceeded 1 mm, the incidence of membrane thickening was 88.2%, and the mean value of thickening was 1.0 mm; however, the variable values of penetration in the 1-mm group were only 37.5% and 0.2 mm, respectively (P \0.05).	penetration depth within 1 mm is recommended for infrazygomatic crest mini-implant anchorage.
Motoyoshi et al., 2015 [23]	A 2 weeks prospective study to investigate the frequency of maxillary sinus perforation after screw placement and the effect of sinus perforation on screw stability.	Maxillary sinus perforations involving 82 miniscrews (diameter, 1.6 mm; length, 8 mm) were evaluated using cone-beam computed tomography. All miniscrews were placed in maxillary alveolar bone between the second premolar and first molar for anchorage for anterior retraction in patients undergoing first premolar extraction. The placement torque and screw mobility of each implant were determined using a torque tester and a Periotest device, and variability in these values in relation to sinus perforation was evaluated.	Eight of the 82 miniscrews perforated the maxillary sinus. There was no case of sinusitis in patients with miniscrew perforation and no significant difference in screw mobility or placement torque between perforating and non-perforating miniscrews. The sinus floor was significantly thinner in perforated cases than in non-perforated cases.	A sinus floor thickness of 6.0 mm or more is recommended in order to avoid miniscrew perforation of the maxillary sinus.

Shinohara et al., 2013 [24]	A 1 month prospective study to investigate root proximity and variability of the placement inclination of a mini-implant according to placement position.	Fifty patients with 147 implants (diameter, 1.6 mm; length, 8 mm) were included. Cone-beam computed tomography images were taken of the area around the implant-placement site. The distances between the root and the mini-implant, and the vertical and horizontal inclinations of the placed implants, were measured.	Of 147 implants, approximately 20% were in contact with a root. The vertical inclinations of the mini-implants were 48.3° to 50.4° in the maxilla and 57.5° to 63.3° in the mandible. In the right maxilla, the incidence of root contact with the distal adjacent tooth was significantly greater than that with the mesial tooth.	One fifth of the mini-implants in this study contacted adjacent roots. During placement of mini-implants in the buccal maxillary right alveolar bone, contact with the root of the distal adjacent tooth should be avoided.
Takaki et al., 2010 [18]	A 9 years retrospective study to determine factors that might cause complications in use of temporary anchorage devices (TADs) for orthodontic anchorage.	In this study were investigated 904 TADs in 455 patients. Clinical diagnoses requiring orthodontic treatment were malocclusion, jaw deformity, various syndromes, cleft lip and palate and impacted teeth. All patients underwent surgery at Tokyo Dental College Chiba Hospital. Three kinds of titanium screw of different diameter and length were used: self-drilling mini-screws (Dual Top Autoscrew® and OSAS®), pre-drilling micro-screws (K1 system®) and palatal screws (PIAS®). Mini-plates fixed with 2 or 3 screws (SAS system®) were	Each type of implant had a high success rate of over about 90%. Failure rates were as follows: micro-screws, 7%; mini-screws, 6%; palatal implants, 11%; and mini-plates, 6%. Inflammation rate occurring in soft tissue surrounding TADs was follows: plate-type, 7.6%; mini-screws, 1.3%; micro-screws, 0%; and palatal implants, 2.5%. Inflammation frequencies depended on degree of mucosal penetration.	Both plate- and screwtype orthodontic implants showed excellent clinical performance.

		also used for skeletal anchorage. Patients were aged between 8 and 68 years (25.7±9.8 years). A total of 460 screw-type and 444 plate-type TADs were used. These comprised the following: mini-plates, 444; self-drilling mini-screws, 225; pre-drilling micro-screws, 83; and palatal screws, 152.	Granulation rate in soft tissue surrounding TADs occurred as follows: micro-screws, 5.7%; self-drilling mini-screws, 0%; palatal screws, 0.6%; plate-type, 0.9%.	
Ziebura et al., 2012 [19]	A 23 months retrospective study to identify insertion procedure and force application related complications in Jet Screw (JS) type mini-implants when a inserted in the palatal slope.	Forty-one consecutively started patients treated using mini-implants in the palatal slope. In this retrospective study, 66 JS were evaluated. Patient records were used to obtain data on the mode of utilization and complications. Standardized photographs overlayed with a virtual grid served to test the hypothesis that deviations from the recommended insertion site or the type of mechanics applied might be related to complications regarding bleeding, gingival overgrowth or implant failure.	Two implants (3%) were lost, and two implants (3%), both loaded with a laterally directed force, exhibited loosening while still serving for anchorage. Complications that required treatment did not occur, the most severe problem observed being gingival proliferation which was attributable neither to patients' age nor to applied mechanics or deviations from the ideal implant position.	Two implants (3%) were lost, and two implants (3%), both loaded with a laterally directed force, exhibited loosening while still serving for anchorage. Complications that required treatment did not occur, the most severe problem observed being gingival proliferation which was attributable neither to patients' age nor to applied mechanics or deviations from the ideal implant position.
Wang et al., 2010 [25]	A 5 years and 7 months retrospective study to examine the incidence of	The factors related to buccal injury during the use of miniscrews were evaluated using	The overall incidence of buccal lesions caused by interradicular miniscrews	Interradicular miniscrews may be associated with damage to the buccal mucosa. For buccal mucosal

	buccal mucosal lesions and identify factors affecting this complication caused by an interradicular miniscrew used as orthodontic anchorage.	the Rank or Fisher exact test. Results were considered significant at P < .05. In all, 136 Aarhus screws in 54 patients were examined.	was 11.8%. The incidences of trauma were 10.4% in men and 12.5% in women, 9.5% in the maxilla and 12.8% in the mandible, 15.0% in patients with a high mandibular plane angle and 9.2% in patients with an average angle, and 28.1% with a miniscrew insertion angle between 10 and 30 degrees, 8.6% with an insertion angle between 30 and 60 degrees, and 4.4% with an insertion angle between 60 and 80 degrees. There were statistically significant differences according to site of placement (P = .00) and occlusogingival position (P = .00).	lesions caused by interradicular miniscrews, site of placement and occlusogingival position are the major risk factors, and the angle of placement and the mandibular plane angle are secondary risk factors. The patient's sex and the arch in which the screw is placed (maxilla versus mandible) have little effect.
Xin et al., 2022 [26]	A 5 years retrospective study to investigate the underlying risk factors influencing the progressive susceptibility of orthodontic miniscrews to failure.	Overall, 889 miniscrews were successively inserted into 347 patients because some loosened or fell off once, twice, or more before achieving their purposes. The number of miniscrew failures (ie, once, twice, or more) was defined as progressive	The progressive susceptibility of miniscrews to failure was proved to be affected by the age of patients, the onset of force application, site of placement, and appliance type. Age and onset of force	Younger people with removable appliances that miniscrews inserted in the retromax- illary or retromandibular regions and earlier onsets of loading had a higher progressive susceptibility to loos- ening. Meanwhile, the failure rate was elevated with the increasing

		susceptibility to failure. The clinical indicators were assessed via univariate analysis, mul- ticollinearity diagnosis, and Poisson log-linear regression model with stepwise calculation to screen out.	application pre- sented a negative relationship with susceptibility. Miniscrews inserted in the palatal region appeared to be more stable than the forepart of the arch. In contrast, the retromaxillary and retromandibular areas obtained the lowest stability. The patients with fixed appliances were more unlikely to suffer progressive failure than removable appliances. In addition, the larger number of screws inserted in each patient, the greater probability of failure.	number of screws per patient received.

Table S7. NHLBI Quality Assessment Tool for Observational Cohort and Cross-Sectional Studies.

	NHLBI Quality Assessment Tool for Observational Cohort and Cross-Sectional Studies														
**First Author et al., Year**	**Q1**	**Q2**	**Q3**	**Q4**	**Q5**	**Q6**	**Q7**	**Q8**	**Q9**	**Q10**	**Q11**	**Q12**	**Q13**	**Q14**	**Total Score**	**Quality Rating**
Fabbroni et al; 2014 ([13])	Y	Y	Y	Y	Y	Y	Y	Y	Y	N	N	N	Y	Y	11/14 (78,57%)	Good
Gurdan et al; 2018 ([14])	Y	Y	Y	Y	N	Y	Y	Y	Y	N	Y	N	Y	N	10/14 (71,43%)	Fair
Hourfar et al; 2017 ([15])	Y	Y	Y	Y	N	Y	Y	Y	Y	N	Y	Y	Y	N	11/14 (78,57%)	Good
Jung et al; 2015 ([16])	Y	Y	Y	Y	N	N	Y	Y	Y	N	Y	Y	N	Y	10/14 (71,43%)	Fair
Jia et al; 2018 ([17])	Y	Y	Y	Y	N	Y	Y	Y	Y	N	Y	Y	Y	N	11/14 (78,57%)	Good
Takaki et al; 2010 ([18])	Y	Y	Y	Y	N	Y	Y	Y	Y	N	Y	Y	Y	N	11/14 (78,57%)	Good
Ziebura et al; 2012 ([19])	Y	Y	Y	Y	N	Y	Y	Y	Y	N	Y	Y	Y	N	11/14 (78,57%)	Good

Q1: Was the research question or objective in this paper clearly stated?, Q2: Was the study population clearly specified and defined?, Q3: Was the participation rate of eligible persons at least 50%?, Q4: Were all the subjects selected or recruited from the same or similar populations (including the same time period)? Were inclusion and exclusion criteria for being in the study prespecified and applied uniformly to all participants?, Q5: Was a sample size justification, power description, or variance and effect estimates provided?, Q6: For the analyses in this paper, were the exposure(s) of interest measured prior to the outcome(s) being measured?, Q7: Was the timeframe sufficient so that one could reasonably expect to see an association between exposure and outcome if it existed?, Q8: For exposures that can vary in amount or level, did the study examine different levels of the exposure as related to the outcome (e.g., categories of exposure, or exposure measured as continuous variable)?, Q9: Were the exposure measures (independent variables) clearly defined, valid, reliable, and implemented consistently across all study participants?, Q10: Was the exposure(s) assessed more than once over time?, Q11: Were the outcome measures (dependent variables) clearly defined, valid, reliable, and implemented consistently across all study participants?, Q12: Were the outcome assessors blinded to the exposure status of participants?, Q13: Was loss to follow-up after baseline 20% or less?, Q14: Were key potential confounding variables measured and adjusted statistically for their impact on the relationship between exposure(s) and outcome(s)?; Total Score: Number of yes; CD: cannot be determined; NA: not applicable; NR: not reported; N: no; Y: yes. Quality Rating: Poor <50%, Fair 50–75%, Good ≥75%.

References

1. Giudice AL, Rustico L, Longo M, et al. Complications reported with the use of orthodontic mini-screws: A systematic review. *Korean J Orthod*. 2021, 51, 199-216.

2. Papageorgiou SN, Zogakis IP, Papadopoulos MA. Failure rates and associated risk factors of orthodontic miniscrew implants: A meta-analysis. *American Journal of Orthodontics and Dentofacial Orthopedics*. 2012;142.

3. Mohamed AM, Yang Y, Yaosen C, et al. Effect of Operator-Related Factors on Failure Rate of Orthodontic Mini-Implants (OMIS) used as Temporary Anchorage Devices (TAD); Systematic Review. *J Dent Oral Care Med*. 2018, 4, 205.

4. Inchingolo AM, Malcangi G, Costa S, et al. Tooth Complications after Orthodontic Miniscrews Insertion. *Int J Environ Res Public Health*. 2023, 20, 1562.

5. Ramírez-Ossa DM, Escobar-Correa N, Ramírez-Bustamante MA, Agudelo-Suárez AA. An Umbrella Review of the Effectiveness of Temporary Anchorage Devices and the Factors That Contribute to Their Success or Failure. *Journal of Evidence-Based Dental Practice*. 2020;20.

6. Kakali L, Alharbi M, Pandis N, Gkantidis N, Kloukos D. Success of palatal implants or mini-screws placed median or paramedian for the reinforcement of anchorage during orthodontic treatment: a systematic review. *European journal of orthodontics,* 2019:9-20.

7. Yi J, Ge M, Li M, et al. Comparison of the success rate between self-drilling and self-tapping miniscrews: a systematic review and meta-analysis. *European Journal of Orthodontics*. 2017, 39, 287-293.

8. Tsui W, Chua H, surgery LC of oral and maxillofacial, 2012 undefined. Bone anchor systems for orthodontic application: a systematic review. *Int J Oral Maxillofac Surg*. 2012, 41, 1427-1438.

9. Mousa MM, Hajeer MY, Sultan K, et al. Evaluation of the Patient-Reported Outcome Measures (PROMs) With Temporary Skeletal Anchorage Devices in Fixed Orthodontic Treatment: A Systematic Review. *Cureus*. 2023, 15, 361-365.10.

10. Khlef H.N., Hajeer M.Y., Ajaj M.A., Heshmeh O. Evaluation of Treatment Outcomes of *En masse* Retraction with Temporary Skeletal Anchorage Devices in Comparison with Two-step Retraction with Conventional Anchorage in Patients with Dentoalveolar Protrusion: A Systematic Review and Meta-analysis. *Contemp Clin Dent*. 2018, 9, 513-523.

11. Gintautaitė G, Gaidytė A. Surgery-related factors affecting the stability of orthodontic mini-implants screwed in alveolar process interdental spaces: a systematic literature review. *Stomatologija*. 2017, 19, 10-18.

.

12. Aromataris E., Munn, Z. JBI Manual for Evidence Synthesis. 2020. Available online: https://synthesismanual.jbi.global (accessed on 30 July 2025).

13. Fabbroni G, Aabed S, Mizen K, Starr DG. Transalveolar screws and the incidence of dental damage: a prospective study. *Int J Oral Maxillofac Surg*. 2004;33:442-446.

14. Gurdan Z, Szalma J. Evaluation of the success and complication rates of self-drilling orthodontic mini-implants. *Niger J Clin Pract*. 2018;21:546-552.

15. Hourfar J, Bister D, Lisson JA, Ludwig B. Incidence of pulp sensibility loss of anterior teeth after paramedian insertion of orthodontic mini-implants in the anterior maxilla. *Head Face Med*. 2017;13:1.

16. Jung SA, Choi YJ, Lee DW, Kim KH, Chung CJ. Cross-sectional evaluation of the prevalence and factors associated with soft tissue scarring after the removal of miniscrews. *Angle Orthod*. 2015;85:420-426.

17. Jia X, Chen X, Huang X. Influence of orthodontic mini-implant penetration of the maxillary sinus in the infrazygomatic crest region. *American Journal of Orthodontics and Dentofacial Orthopedics*. 2018;153:656-661.

18. Takaki T, Tamura N, Yamamoto M, et al. Clinical study of temporary anchorage devices for orthodontic treatment--stability of micro/mini-screws and mini-plates: experience with 455 cases. *Bull Tokyo Dent Coll*. 2010;51:151-163.

19. Ziebura T, Flieger S, Wiechmann D. Mini-implants in the palatal slope - A retrospective analysis of implant survival and tissue reaction. *Head Face Med*. 2012;8.

20. Golshah A, Gorji K, Nikkerdar N. Effect of miniscrew insertion angle in the maxillary buccal plate on its clinical survival: a randomized clinical trial. *Prog Orthod*. 2021;22.

21. Aboshady H, Mohamed A, Abouelezz A, et al. Failure Rate of Orthodontic Mini-screw after Insertion using 3D Printed Guide versus Conventional Free Hand Placement Technique: Split Mouth Randomized Clinical Trial. *Open Access Maced J Med Sci*. 2022;10:6-13.

22. Fäh R, Schätzle M. Complications and adverse patient reactions associated with the surgical insertion and removal of palatal implants: a retrospective study. *Clin Oral Implants Res*. 2014, 25, 653-658.

23. Motoyoshi M, Sanuki-Suzuki R, Uchida Y, et al. Maxillary sinus perforation by orthodontic anchor screws. *J Oral Sci*. 2015, 57, 95-100.

24. Shinohara A, Motoyoshi M, Uchida Y, et al. Root proximity and inclination of orthodontic mini-implants after placement: cone-beam computed tomography evaluation. *Am J Orthod Dentofacial Orthop*. 2013, 144, 50-56.

25. Wang Z, Zhang D, Liu Y, et al. Buccal mucosal lesions caused by the inter radicular mini-screw: a preliminary report. *Int J Oral Maxillofac Implants.* 2010, 25, 1183-1188.

26. Xin Y, Wu Y, Chen C, et al. Miniscrews for orthodontic anchorage: analysis of risk factors correlated with the progressive susceptibility to failure. *Am J Orthod Dentofacial Orthop*. 2022, 162, 192-202.
